# A new genus of air-breathing marine slugs from South-East Asia (Gastropoda, Pulmonata, Onchidiidae)

**DOI:** 10.3897/zookeys.877.36698

**Published:** 2019-09-02

**Authors:** Benoît Dayrat, Tricia C. Goulding, Munawar Khalil, Joseph Comendador, Quảng Ngô Xuân, Siong Kiat Tan, Shau Hwai Tan

**Affiliations:** 1 Department of Biology, Pennsylvania State University, University Park, PA 16802, USA; 2 Bernice Pauahi Bishop Museum, Malacology, 1525 Bernice St, Honolulu, HI 96817, USA; 3 Department of Marine Science, Universitas Malikussaleh, Reuleut Main Campus, Kecamatan Muara Batu, North Aceh, Aceh, 24355, Indonesia; 4 National Museum of the Philippines, Taft Ave, Ermita, Manila, 1000 Metro Manila, Philippines; 5 Institute of Tropical Biology, Vietnam Academy of Science and Technology, Ho Chi Minh city, Vietnam; 6 Graduate University of Science and Technology, Vietnam Academy of Science and Technology, Hanoi, Vietnam; 7 Lee Kong Chian Natural History Museum, 2 Conservatory Dr, National University of Singapore, 117377, Singapore; 8 School of Biological Sciences, Universiti Sains Malaysia, 11800 Penang, Malaysia; 9 Centre for Marine and Coastal Studies, Universiti Sains Malaysia, 11800 Penang, Malaysia

**Keywords:** Biodiversity, biogeography, Euthyneura, integrative taxonomy, revisionary systematics

## Abstract

As part of an ongoing effort to revise the taxonomy of air-breathing, marine, onchidiid slugs, a new genus, *Laspionchis* Dayrat & Goulding, **gen. nov.**, is described from the mangroves of South-East Asia. It includes two new species, *Laspionchis boucheti* Dayrat & Goulding, **sp. nov.**, and *Laspionchis bourkei* Dayrat & Goulding, **sp. nov.**, both distributed from the Malacca Strait to the Philippines and Australia. This study is based on extensive field work in South-East Asia, comparative anatomy, and both mitochondrial (COI and 16S) and nuclear (ITS2 and 28S) DNA sequences. The two new species are found in the same habitat (mud surface in mangrove forests) and are externally cryptic but are distinct anatomically. Both species are also strongly supported by DNA sequences. Three cryptic, least-inclusive, reciprocally-monophyletic units within *Laspionchis bourkei* are regarded as subspecies: *L. bourkei bourkei* Dayrat & Goulding, **ssp. nov.**, *L. bourkei lateriensis* Dayrat & Goulding, **ssp. nov.**, and *L. bourkei matangensis* Dayrat & Goulding, **ssp. nov.** The present contribution shows again that species delineation is greatly enhanced by considering comparative anatomy and nuclear DNA sequences in addition to mitochondrial DNA sequences, and that thorough taxonomic revisions are the best and most efficient path to accurate biodiversity knowledge.

## Introduction

The diversity of invertebrate species in mangrove forests of South-East Asia is still largely unknown, mainly because mangroves have not been explored well enough, which likely has to do with the fact that mangroves are not the most inviting habitats, even for savvy field naturalists: mangroves are extremely muddy, infested with malaria-carrying mosquitoes and pit vipers, and often located in remote areas. Our lack of biodiversity knowledge is a major issue not only because nobody knows exactly how many species live in the mangroves of South-East Asia, but also because mangroves are still being eradicated at a large scale across the entire region. Onchidiid slugs illustrate well this general situation: until recently, nobody knew exactly how many species of onchidiids lived in the mangroves of South-East Asia, even though they are some of the most common and diverse animals in mangroves (Dayrat 2009).

Onchidiids are marine, air-breathing, true slugs. Adult onchidiids live in the intertidal zone and their larvae develop in sea water, although a few species are adapted to high elevation tropical rainforest (Dayrat 2010). They breathe air through a lung and are related to land snails and slugs (Dayrat et al. 2011). They are called 'true' slugs because they lack an internal shell, even a vestigial shell. The only other group of marine, air-breathing, true slugs is the genus *Smeagol* Climo, 1980, which is not closely-related to onchidiids, but rather considered to belong to the Ellobiidae (Dayrat et al. 2011). The terrestrial, air-breathing, true veronicellid slugs are the most-closely related group to the onchidiids (Dayrat et al. 2011).

In the past ten years, our laboratory has worked on a global taxonomic revision of the Onchidiidae, one genus at a time (Dayrat et al. 2016, 2017, 2018, 2019; Dayrat and Goulding 2017; Goulding et al. 2018a, b, c), based on extensive collecting efforts worldwide, especially in South-East Asia where onchidiids have greatly diversified. The application of old generic names, such as *Onchidium* Buchannan, 1800, *Onchidina* Semper, 1882, *Paraoncidium* Labbé, 1934, and *Peronina* Plate, 1893, is now clear, and new genera are also being discovered: *Alionchis* Goulding & Dayrat in Goulding et al. 2018b, *Marmaronchis* Dayrat & Goulding in Dayrat et al. 2018, *Melayonchis* Dayrat & Goulding in Dayrat et al. 2017, *Paromoionchis* Dayrat & Goulding in Dayrat et al. 2019, and *Wallaconchis* Goulding & Dayrat in Goulding et al. 2018a.

In the present contribution, we describe a new genus, *Laspionchis* gen. nov., and two new species: *Laspionchis boucheti* sp. nov., distributed from the Malacca Strait eastwards to the Philippines and Queensland, Australia, and *Laspionchis bourkei* sp. nov., from the Malacca Strait eastwards to the Philippines and the Northern Territory, Australia. Three cryptic, least-inclusive, reciprocally-monophyletic units within *Laspionchis bourkei* are regarded as three subspecies: *L. bourkei bourkei* Dayrat & Goulding, ssp. nov., *L. bourkei lateriensis* Dayrat & Goulding, ssp. nov., and *L. bourkei matangensis* Dayrat & Goulding, ssp. nov. New taxon names are needed because no existing genus-group name applies to the clade described here and no existing species-group name applies to the species and subspecies described here.

The present study follows an integrative approach to taxonomy (Dayrat 2005), which is based on: (1) a comprehensive review of the nomenclature (all available types of onchidiid species were borrowed and re-examined); (2) the field observation of live animals in their natural habitats; (3) comparative anatomy; and (4) analyses of both mitochondrial (COI, 16S) and nuclear (ITS2, 28S) DNA sequences. The new genus described here is characterized by a combination of anatomical characters which is unique (not found in other onchidiid genera), and its monophyly is strongly supported in molecular phylogenetic analyses. Even though both species are cryptic externally, they are strongly supported by DNA sequences and internal anatomy.

*Laspionchis* slugs live on the surface of the mud in mangrove forests, where they co-occur with many other onchidiid species with a similar appearance. *Laspionchis* slugs are most especially difficult to distinguish externally from *Paromoionchis* slugs, which are found in the same habitats and the same geographical regions (Dayrat et al. 2019).

## Materials and methods

### Collecting

All specimens were collected by the authors in the last few years. Collecting parties were led by Benoît Dayrat in Brunei Darussalam, Malaysia, Northern Territory (Australia), Philippines, and Singapore, by Tricia Goulding in Queensland (Australia) and Vietnam, and by Munawar Khalil in Indonesia. We often were accompanied by local villagers or fishermen. Sites were accessed by car or by boat. Each site was explored for an average of two hours, but the exact time spent at each site also depended on the time of the low tide, the weather, etc. At each site, photographs were taken to document the kind of mangrove being visited as well as the diverse microhabitats where specimens were collected.

In the field, specimens were individually numbered and photographed in their habitat. At each site, we tried our best to sample as much diversity as possible. In addition to numbering individually the specimens that looked different, we also numbered individually many specimens that looked similar so that we could test for the presence of cryptic diversity. Importantly, a piece of tissue was cut for all specimens individually numbered (for DNA extraction) and the rest of each specimen was relaxed (using magnesium chloride) and fixed (using 10% formalin or 70% ethanol) for comparative anatomy.

### Specimens

All available types of Onchidiidae were examined. Many worldwide museum collections were visited (but no *Laspionchis* material was found). Sixty-one specimens of *Laspionchis* are included in this study: 23 specimens of *L. boucheti* and 38 specimens of *L. bourkei*. Each specimen was examined for comparative anatomy and sequenced for molecular phylogenetic analyses. Individual DNA extraction numbers used in the phylogenetic analyses are indicated in the lists of material examined (numbers are between brackets, and a capitalized letter H indicates a holotype), and size (length/width) is indicated in millimeters (mm) for each specimen. All specimens were deposited as vouchers in institutions in the countries of origin.

### Museum collection abbreviations

**BDMNH** Brunei Museum, Natural History, Brunei Darussalam

**ITBZC** Institute of Tropical Biology, Zoology Collection, Vietnam Academy of Science and Technology, Ho Chi Minh City, Vietnam

**MTQ** Museum of Tropical Queensland, Townsville, Queensland, Australia

**NTM** Museum and Art Gallery of the Northern Territory

**PNM** National Museum of the Philippines, Manila, Philippines

**UMIZ** Universitas Malikussaleh, North Aceh, Sumatra, Indonesia

**USMMC** Universiti Sains Malaysia, Mollusk Collection, Penang, Malaysia

**ZRC** Zoological Reference Collection, Lee Kong Chian Natural History Museum, National University of Singapore

### Anatomical preparations and descriptions

Both the external morphology and the internal anatomy were studied. All anatomical observations were made under a dissecting microscope and drawn with a camera lucida. Radulae and male reproductive organs were prepared for scanning electron microscopy (Zeiss SIGMA Field Emission Scanning Electron Microscopy). Radulae were cleaned in 10% NaOH for a week, rinsed in distilled water, briefly cleaned in an ultrasonic water bath (less than a minute), sputter-coated with gold-palladium, and examined by SEM. Soft parts (penis, accessory penial gland, etc.) were dehydrated in ethanol and critical point dried before coating.

The anatomy of *L. boucheti*, the type species, is fully detailed. The written description of the many anatomical features that are virtually identical between species (nervous system, heart, etc.) is given only for the type species to avoid repetition. So, any feature that is only mentioned in *L. boucheti* is identical in the other species. The color of live animals is described in detail for both species in order to demonstrate the overlapping individual variation between species. As expected, differences between species are mostly found in the male copulatory apparatus, which is described and illustrated in detail for each species. Special attention has been paid to illustrating the holotype of each of the species and subspecies, and the plates illustrating habitats also include a picture from type localities.

### Intestinal types

Now that the types of intestinal loops have been reported for every species in many genera of onchidiids (Dayrat et al. 2016, 2017, 2018, 2019;Dayrat and Goulding 2017; Goulding et al. 2018a, b, c), it is possible, and actually necessary, to clarify the differences between the various types of intestinal loops. Plate (1893: pl. 8, figs 29–32) first distinguished four types of intestinal loops (types I to IV) and Labbé (1934: 177–178, fig. 3) later added a type V. However, the pattern of intestinal loops varies, both intra-specifically and inter-specifically. The differences between intestinal types are not as sharp as Plate and Labbé assumed they were, and now they must be clarified.

**Figure 1.**
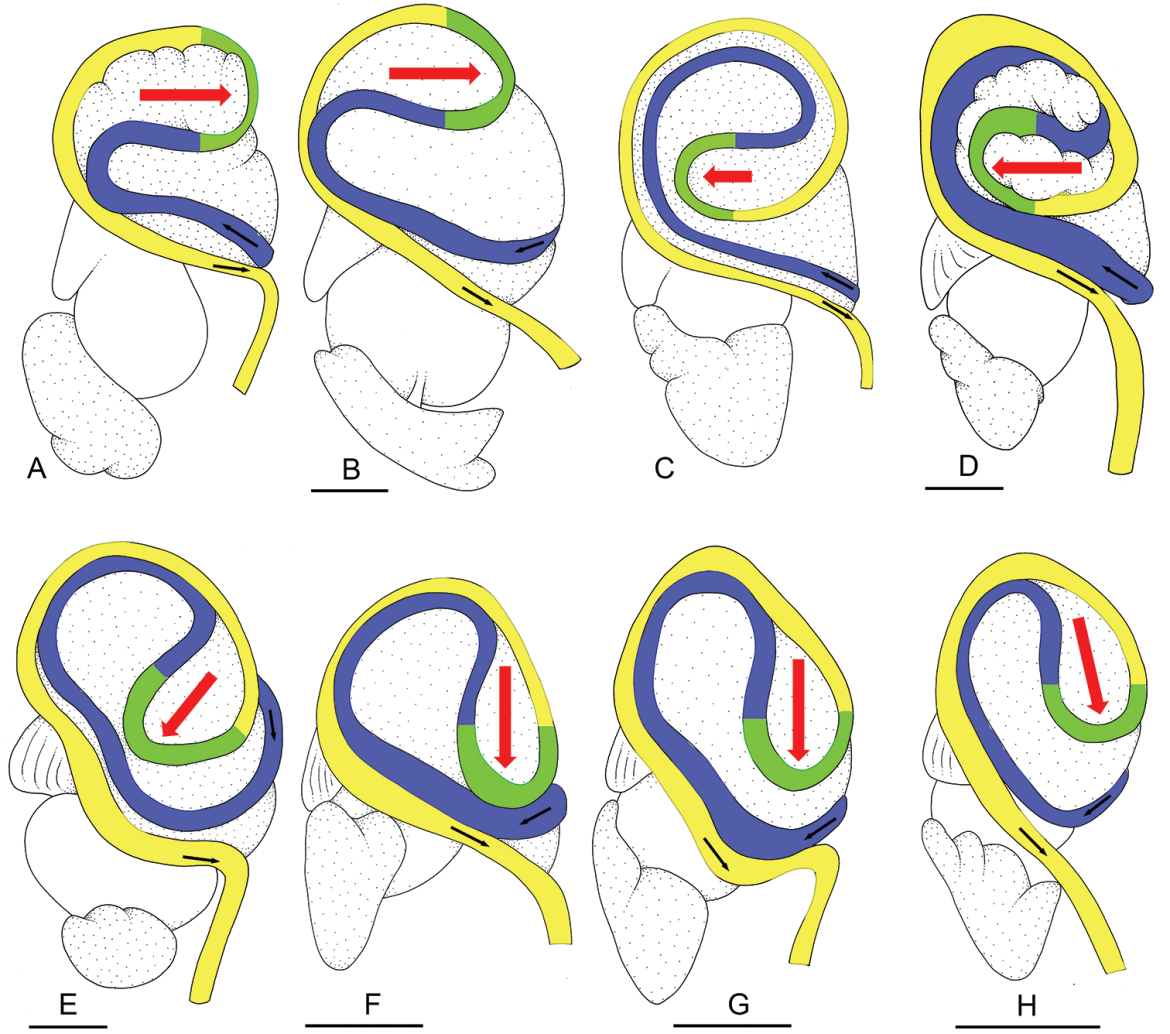
Intestinal loops, dorsal view **A, B** Type I **C, D** Type II **E–H** Between types I and II. Small black arrows show the direction of the intestinal transport. The clockwise loop is in blue. The counterclockwise loop is in yellow. The transitional loop (between clockwise and counterclockwise loops) is in green. Red arrows indicate the orientation of the transitional loop. **A** Type I, with a transitional loop oriented at 3 o’clock, redrawn from Plate (1893: pl. 8, fig. 29) **B** Type I, with a transitional loop oriented at 3 o’clock, *Wallaconchis sinanui* (from Goulding et al. 2018: fig. 8D) **C** Type II, with a transitional loop oriented at 9 o’clock, redrawn from Plate (1893: pl. 8, fig. 31) **D** Type II, with a transitional loop oriented at 9 o’clock, *Paromoionchis tumidus* (from Dayrat et al. 2019: fig. 12A) **E** Between types I and II, with a descending transitional loop at 7 o’clock, holotype, *Laspionchis boucheti*, Australia, Northern Territory, [1688 H] (NTM P.57614) **F** Between types I and II, with a descending transitional loop at 6 o’clock, *L. boucheti*, Australia, Northern Territory, [1681] (NTM P.57612) **G** Between types I and II, with a descending transitional loop at 6 o’clock, *L. boucheti*, Vietnam, [5610] (ITBZC IM 00017) **H** Between types I and II, with a descending transitional loop at 5 o’clock, *L. boucheti*, Australia, Queensland, [2612] (MTQ). Scale bars: 1 mm (**B**), 2 mm (**D, E**), 5 mm (**F–H)**.

Here we provide a new approach to help reliably determine intestinal types. Because the intestinal loops found in *Laspionchis* are between type I and type II, we focus here on types I and II. This new approach is based on recognizing three different sections in intestinal loops, each section being colored differently: a clockwise loop is colored in blue, a counterclockwise loop in yellow, and a transitional loop between them in green (Fig. 1). For the sake of clarity, Plate’s (1893: pl. 8, figs 29, 31) original illustrations of his types I and II are reproduced here (Fig. 1A, C).

The onchidiid types of intestinal loops are defined based on the dorsal pattern of the intestine. The intestine always first appears dorsally on the right side. In a type I (Fig. 1A, B), the intestine starts by forming a clockwise loop (blue loop). This clockwise loop, however, does not form a complete circle and soon transitions into a counterclockwise loop (yellow loop). As a result, the transitional loop (green loop) between the clockwise and counterclockwise loops is oriented to the right, at 3 o’clock (horizontal red arrow). In a type II (Fig. 1C, D), the clockwise loop (blue loop) is longer and rotates more than in a type I and, as a result, the transitional (green) loop is oriented to the left at 9 o’clock (horizontal red arrow). There is, as always, individual variation. Most usually, the orientation of the transitional loop varies within a range of ca. 90 degrees around a mean axis (i.e., 45 degrees on either side of that axis). So, for instance, in *Paromoionchis tumidus* (Semper, 1880), the species that illustrates a typical type II (Fig. 1D), the transitional loop is always oriented to the left but is not always perfectly horizontal (at 9 o’clock); it can be descending (down to approximately 7 o’clock) or ascending (up to approximately 11 o’clock) (Dayrat et al. 2019: fig. 12).

In *Laspionchis* (Fig. 1E–H), the average orientation of the transitional loop is exactly between a typical type I and a typical type II. Indeed, in most *Laspionchis* individuals, the transitional loop is descending vertically at 6 o’clock (Fig. 1F, G). So, intestinal loops of *Laspionchis* slugs cannot be assigned to either type I or type II. Naturally, there is individual variation (Fig. 1E–H): every specimen listed in the material examined was dissected to check its intestinal loops. In some cases, the intestinal loops appear to be of type II, with the transitional loop oriented to the left and descending at approximately 7 o’clock (see the red arrow in Fig. 1E). In some other cases, the intestinal loops appear to be of type I, with the transitional loop oriented to the right and descending at approximately 5 o’clock (see the red arrow in Fig. 1H). In the individuals examined for the present study, the transitional loop is not higher than 5 o'clock on the right (i.e., it is not oriented at 4 or 3 o’clock) and not higher than 7 o’clock on the left (i.e., it is not oriented at 8 or 9 o’clock). So, the intestinal loops of the two known species of *Laspionchis* are exactly between types I and II. Instead of creating a new intestinal type (number VI), the intestinal loops of *Laspionchis* are simply and adequately referred to as “between types I and II.”

### DNA extraction and PCR amplification

DNA was extracted using a phenol-chloroform extraction protocol with cetyltrimethyl-ammonium bromide (CTAB). The mitochondrial cytochrome *c* oxidase I region (COI) and 16S region were amplified using the following universal primers: LCO1490 (5'-3') GGT CAA CAA ATC ATA AAG ATA TTG G, HCO2198 (5'-3') and TAA ACT TCA GGG TGA CCA AAR AAY CA (Folmer et al. 1994), 16Sar-L (5'-3') CGC CTG TTT ATC AAA AAC AT (Palumbi 1996), and the modified Palumbi primer 16S 972R (5'-3') CCG GTC TGA ACT CAG ATC ATG T (Dayrat et al. 2011). The nuclear ITS2 region and 28S region were amplified with the following primers: LSU-1 (5'-3') CTA GCT GCG AGA ATT AAT GTG A, LSU-3 (5'-3') and ACT TTC CCT CAC GGT ACT TG (Wade and Mordan 2000), 28SC1 (5'-3') ACC CGC TGA ATT TAA GCA T (Hassouna et al. 1984), and 28SD3 (5'-3') GAC GAT CGA TTT GCA CGT CA (Vonnemann et al. 2005). The 25 μl PCRs for COI and 16S contained 15.8 μl of water, 2.5 μl of 10× PCR Buffer, 1.5 μl of 25 mM MgCl_2_, 0.5 μl of each 10 μM primer, 2 μl of dNTP Mixture, 0.2 μl (1 unit) of TaKaRa Taq (Code No. R001A), 1 μl of 20 ng/μl template DNA, and 1 μl of 100× BSA (Bovine Serum Albumin). The PCRs for ITS2 used the reagents in the same amounts as COI and 16S, except that water was reduced to 14.8 μl and the amount of 100× BSA was increased to 2 μl. The PCR reaction for 28S included 14.8 μl of water, 2.5 μl of 10× PCR Buffer, 0.5 μl of each 10 μM primer, 1 μl of dNTP Mixture, 5 μl of Q solution (which includes MgCl_2_) and 0.5 μl of 20 ng/μl template DNA. The thermoprofile used for COI and 16S was: 5 minutes at 94 °C; 30 cycles of 40 seconds at 94 °C, 1 minute at 46 °C, and 1 minute at 72 °C; and a final extension of 10 minutes at 72 °C. The thermoprofile used for ITS2 was: 1 minute at 96 °C; 35 cycles of 30 seconds at 94 °C, 30 seconds at 50 °C, and 1 minute at 72 °C; and a final extension of 10 minutes at 72 °C. The thermoprofile used for 28S was: 4 minutes at 94 °C; 38 cycles of 50 seconds at 94 °C, 1 minute at 52 °C, and 2 minutes 30 seconds at 72 °C; and a final extension of 10 minutes at 72 °C. The PCR products were cleaned with ExoSAP-IT (Affymetrix, Santa Clara, CA, USA) prior to sequencing. Untrimmed sequenced fragments represented approximately 680 bp for COI, 530 bp for 16S, 740 bp for ITS2, and 1000 bp for 28S.

### Phylogenetic analyses

Chromatograms were consulted to resolve rare ambiguous base calls. DNA sequences were aligned using Clustal W in MEGA 6 (Tamura et al. 2013). Nineteen onchidiid species outside *Laspionchis* were selected as outgroups from our previous studies (Dayrat et al. 2011, 2016, 2017, 2018, 2019; Dayrat and Goulding 2017; Goulding et al. 2018a, b, c): *Alionchis jailoloensis* Goulding & Dayrat in Goulding et al. 2018b, *Marmaronchis marmoratus* (Lesson, 1831), *Marmaronchis vaigiensis* (Quoy & Gaimard, 1825), *Melayonchis aileenae* Dayrat & Goulding in Dayrat et al. 2017, *Melayonchis eloisae* Dayrat in Dayrat et al. 2017, *Onchidella celtica* (Cuvier in Audouin & Milne-Edwards, 1832), *Onchidella nigricans* (Quoy & Gaimard, 1832), *Onchidina australis* (Semper, 1880), *Onchidium stuxbergi* (Westerlund, 1883), *Onchidium typhae* Buchannan, 1800, *Paromoionchis daemelii* (Semper, 1880), *Paromoionchis tumidus* (Semper, 1880), *Peronia* sp. (Hawaii), *Peronia* sp. (Okinawa), *Peronina tenera* (Stoliczka, 1869), *Peronina zulfigari* Goulding & Dayrat in Goulding et al. 2018c, *Platevindex luteus* (Semper, 1880), *Wallaconchis ater* (Lesson, 1831), and *Wallaconchis sinanui* Goulding & Dayrat in Goulding et al. 2018a. DNA sequences were all deposited in GenBank and vouchers deposited in museum collections (Table 1). The ends of each alignment were trimmed. Alignments of mitochondrial (COI and 16S) sequences and nuclear (ITS2 and 28S) sequences were concatenated separately, in order to test whether these two data sets support the same relationships. The concatenated mitochondrial alignment included 991 nucleotide positions: 577 (COI) and 414 (16S). The concatenated ITS2 and 28S alignment included 1614 nucleotide positions: 658 (ITS2) and 956 (28S).

**Table 1.** GenBank accession numbers for COI, 16S, ITS2, and 28S DNA sequences. All sequences are new, except the sequences of the outgroups which were obtained from our previous studies (Dayrat et al. 2011, 2016, 2017, 2018, 2019; Dayrat and Goulding 2017; Goulding et al. 2018a, b, c).

Species	Individual (DNA #)	Locality	GenBank COI	GenBank 16S	GenBank ITS2	GenBank 28S
*Alionchis jailoloensis*	5137	Halmahera, Indonesia	MG953528	MG953538	MG953548	MK122918
*Marmaronchis marmoratus*	5409	Madang, Papua New Guinea	MK122838	MK122859	MK122893	MK122915
*Marmaronchis vaigiensis*	1183	Singapore	MK122812	MK122854	MK122877	MK122910
*Melayonchis aileenae*	970	Peninsular Malaysia	KX240033	KX240057	MK122902	MK125514
*Melayonchis eloisae*	1011	Singapore	KX240026	KX240050	MK122904	MK125515
*Onchidella celtic*a	5013	France	MG958715	MG958717	MK122906	MK122921
*Onchidella nigricans*	1524	New South Wales, Australia	MG970878	MG970944	MK122908	MK122923
*Onchidina australis*	1523	New South Wales, Australia	KX179548	KX179561	MG958719	MG958887
*Onchidium stuxbergi*	5605	Vietnam	KX179520	KX179537	MG958721	MG958886
*Onchidium typhae*	965	Peninsular Malaysia	KX179509	KX179525	MG958720	MG958885
*Paromoionchis daemelii*	1511	New South Wales, Australia	MH055048	MH055129	MH055241	MH055289
*Paromoionchis tumidus*	1732	Sumatra, Indonesia	MH054951	MH055104	MH055196	MH055268
*Peronia* sp.	696	Okinawa, Japan	HQ660043	HQ659911	MG958871	MG958883
*Peronia* sp.	706	Hawaii, USA	HQ660038	HQ659906	MG958722	MG958884
*Peronina tenera*	960	Peninsular Malaysia	MG958740	MG958796	MG958840	MG958874
*Peronina zulfigari*	6005	Peninsular Malaysia	MG958775	MG958831	MG958867	MG958882
*Platevindex luteus*	1001	Singapore	MG958714	MG958716	MG958718	MG958888
*Wallaconchis ater*	5121	Halmahera, Indonesia	MG970820	MG970911	MG971134	MG971186
*Wallaconchis sinanui*	2740	Ambon, Indonesia	MG970713	MG970881	MG971093	MG971161
***L. boucheti***	1004	Singapore	MH619242	MH619303		
	1005	Singapore	MH619243	MH619304	MH619364	MH619413
	1037	Brunei Darussalam	MH619244	MH619305	MH619365	
	1038	Brunei Darussalam	MH619245	MH619306	MH619366	MH619414
	1679	Northern Territory, Australia	MH619246	MH619307		
	1681	Northern Territory, Australia	MH619247	MH619308	MH619368	MH619416
	1685	Northern Territory, Australia	MH619248	MH619309		
	1688 H	Northern Territory, Australia	MH619249	MH619310		
	1729	Sumatra, Indonesia	MH619250	MH619311	MH619369	
	2559	Queensland, Australia	MH619251	MH619312	MH619370	
	2578	Queensland, Australia	MH619252	MH619313	MH619371	
	2593	Queensland, Australia	MH619253	MH619314	MH619372	
	2604	Queensland, Australia	MH619254	MH619315	MH619373	MH619417
	2609	Queensland, Australia	MH619255	MH619316	MH619374	MH619418
	2612	Queensland, Australia	MH619256	MH619317	MH619375	
	2692	Queensland, Australia	MH619257	MH619318	MH619376	MH619419
	2693	Queensland, Australia	MH619258	MH619319		
	3615	Bohol, Philippines	MH619259	MH619320	MH619377	MH619420
	914	Peninsular Malaysia	MH619260	MH619321	MH619378	MH619421
	915	Peninsular Malaysia	MH619261	MH619322	MH619379	
	5520	Peninsular Malaysia	MH619262	MH619323	MH619380	
	5609	Vietnam	MH619263	MH619324	MH619381	
	5610	Vietnam	MH619264	MH619325	MH619382	MH619422
***L. bourkei bourkei***	1656	Northern Territory, Australia	MH619290	MH619351		
	1616	Northern Territory, Australia	MH619291	MH619352	MH619402	MH619432
	1617	Northern Territory, Australia	MH619292	MH619353	MH619403	
	1618	Northern Territory, Australia	MH619293	MH619354		
	1621	Northern Territory, Australia	MH619294	MH619355	MH619404	
	1652	Northern Territory, Australia	MH619295	MH619356	MH619405	
	1657 H	Northern Territory, Australia	MH619296	MH619357	MH619406	
	1659	Northern Territory, Australia	MH619297	MH619358	MH619407	
	1666	Northern Territory, Australia	MH619298	MH619359	MH619408	
	1673	Northern Territory, Australia	MH619299	MH619360	MH619409	MH619434
	1692	Northern Territory, Australia	MH619300	MH619361	MH619410	
	1693	Northern Territory, Australia	MH619301	MH619362	MH619411	MH619435
	1694	Northern Territory, Australia	MH619302	MH619363	MH619412	MH619436
***L. bourkei lateriensis***	2743	Ambon, Indonesia	MH619284	MH619345	MH619396	MH619429
	2753	Ambon, Indonesia	MH619285	MH619346	MH619397	MH619430
	6061	Ambon, Indonesia	MH619286	MH619347	MH619398	
	6063	Ambon, Indonesia	MH619287	MH619348	MH619399	
	6064 H	Ambon, Indonesia	MH619288	MH619349	MH619400	MH619431
	6065	Ambon, Indonesia	MH619289	MH619350	MH619401	
***L. bourkei matangensis***	978	Singapore	MH619265	MH619326	MH619383	MH619423
	979	Singapore	MH619266	MH619327	MH619384	MH619424
	980	Singapore	MH619267	MH619328		
	983	Singapore	MH619268	MH619329		
	985	Singapore	MH619269	MH619330		
	1783	Sumatra, Indonesia	MH619270	MH619331		
	1784	Sumatra, Indonesia	MH619271	MH619332		
	1785	Sumatra, Indonesia	MH619272	MH619333	MH619385	
	2230	Sulawesi, Indonesia	MH619273	MH619334	MH619386	MH619425
	3343	Bohol, Philippines	MH619274	MH619335	MH619387	MH619426
	3616	Bohol, Philippines	MH619275	MH619336	MH619388	
	5627	Vietnam	MH619276	MH619337	MH619389	MH619427
	5646	Vietnam	MH619277	MH619338	MH619390	
	5958 H	Peninsular Malaysia	MH619278	MH619339	MH619391	MH619428
	5959	Peninsular Malaysia	MH619279	MH619340	MH619392	
	5960	Peninsular Malaysia	MH619280	MH619341	MH619393	
	5961	Peninsular Malaysia	MH619281	MH619342	MH619394	
	5963	Peninsular Malaysia	MH619282	MH619343	MH619395	
	5965	Peninsular Malaysia	MH619283	MH619344		

Four independent sets of phylogenetic analyses were performed: 1) Maximum Likelihood and Bayesian analyses with concatenated mitochondrial COI and 16S sequences; 2) Maximum Likelihood and Bayesian analyses with concatenated nuclear ITS2 and 28S sequences; 3) Maximum Parsimony analyses with concatenated nuclear ITS2 and 28S sequences; 4) Maximum Parsimony analyses with just nuclear ITS2 sequences. Prior to Maximum Likelihood and Bayesian phylogenetic analyses, the best-fitting evolutionary model was selected for each locus separately using the Model Selection option from Topali v2.5 (Milne et al. 2004): a GTR + G model was independently selected for COI and 16S, and an HKY + G model was independently selected for ITS2 and 28S. Maximum Likelihood analyses were performed using PhyML (Guindon and Gascuel 2003) as implemented in Topali v2.5. Node support was evaluated using bootstrapping with 100 replicates. Bayesian analyses were performed using MrBayes v3.1.2 (Ronquist and Huelsenbeck 2003) as implemented in Topali v2.5, with five simultaneous runs of 1.5×10^6^ generations each, sample frequency of 100, and burn in of 25% (and posterior probabilities were also calculated). Topali did not detect any issue with respect to convergence. Maximum Parsimony analyses were conducted in PAUP v 4.0 (Swofford 2002), with gaps coded as a fifth character state and 100 bootstrap replicates conducted using a full heuristic search. All analyses were run several times and yielded the same result.

In addition, another set of analyses was performed with only COI sequences: genetic distances between COI sequences were calculated in MEGA 6 as uncorrected p-distances. COI sequences were also translated into amino acid sequences in MEGA using the invertebrate mitochondrial genetic code to check for the presence of stop codons (no stop codon was found).

## Results

### Molecular phylogenetic analyses

**Figure 2.**
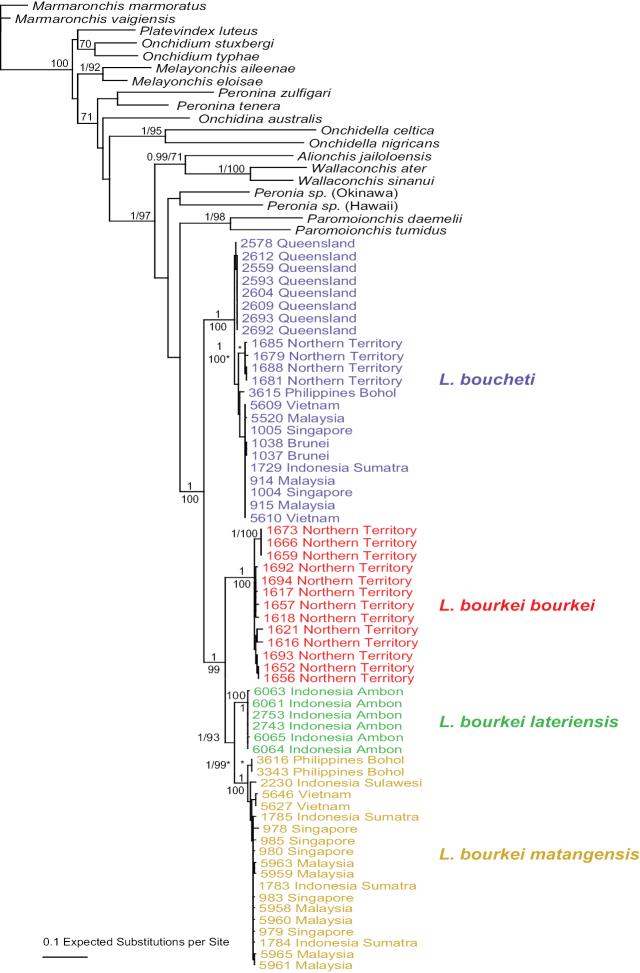
Phylogenetic relationships within *Laspionchis* based on concatenated mitochondrial COI and 16S DNA sequences for 80 individuals (including 19 outgroups). Numbers by the branches are the bootstrap values (maximum likelihood analysis, ML) and the posterior probabilities (Bayesian analysis). Only numbers > 60% (ML) and > 0.9 (Bayesian) are indicated. Numbers for each individual correspond to unique identifiers for DNA extraction. All sequences of *Laspionchis* individuals are new. Sequences of the outgroups are from our previous studies (Dayrat et al. 2011, 2016, 2017, 2018, 2019; Dayrat and Goulding 2017; Goulding et al. 2018a, b, c). Information on specimens can be found in the lists of material examined and in Table 1. The color used for each subspecies is the same as the color used in Figs 3–7.

**Figure 3.**
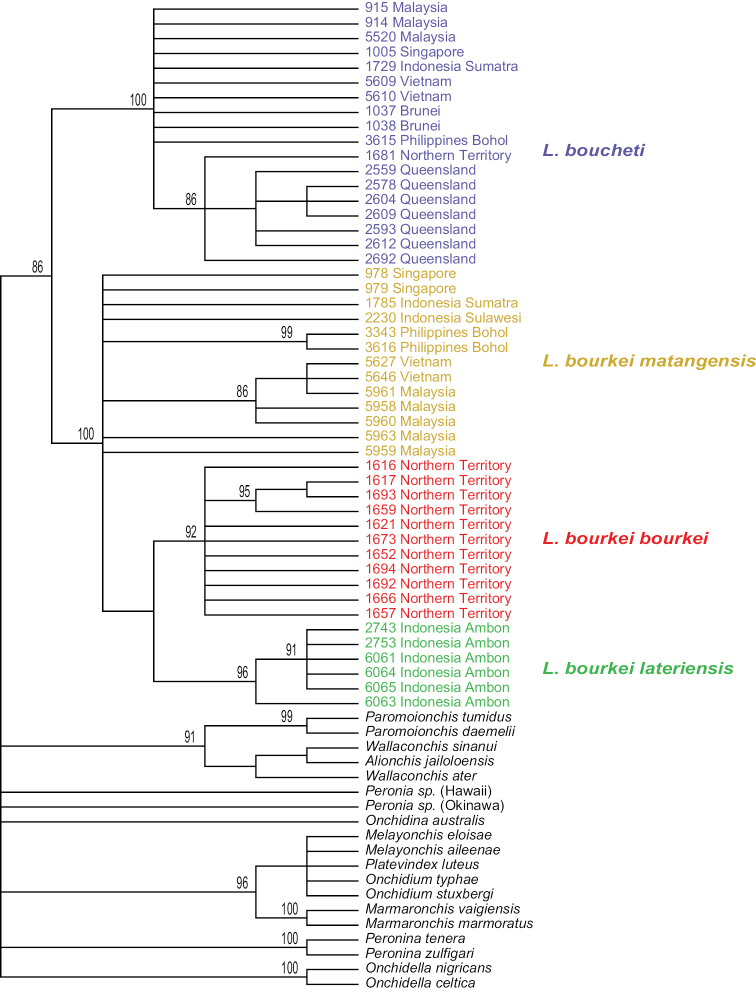
Maximum parsimony consensus tree within *Laspionchis*, performed with ITS2 DNA sequences from 67 individuals (including 19 outgroups). Numbers by the branches are the bootstrap values (only numbers > 70% are indicated). Numbers for each individual correspond to unique identifiers for DNA extraction. All sequences of *Laspionchis* individuals are new. Outgroups sequences are from our previous studies (Dayrat et al. 2011, 2016, 2017, 2018, 2019; Dayrat and Goulding 2017; Goulding et al. 2018a, b, c). Information on specimens can be found in the lists of material examined and in Table 1. The color used for each subspecies is the same as the color used in Figs 2, 4–7.

**Figure 4.**
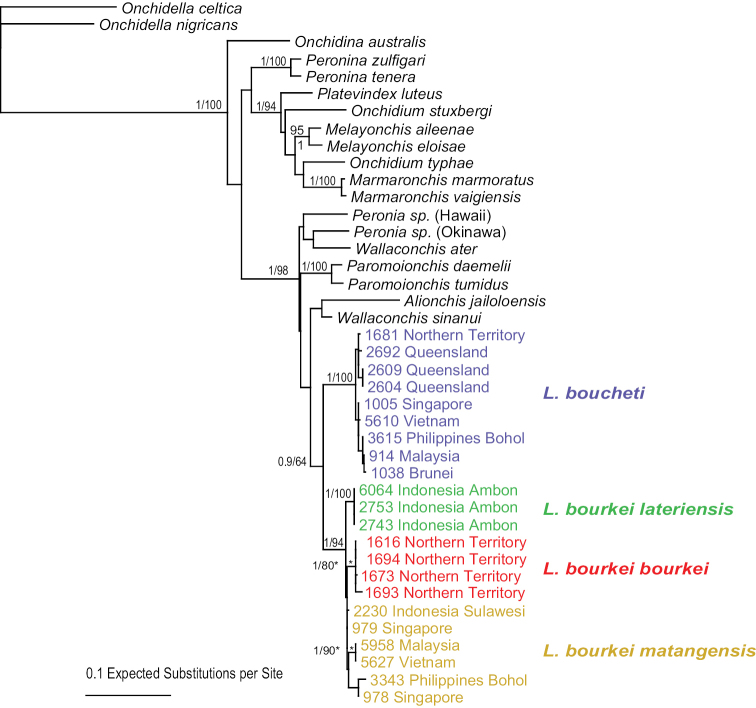
Phylogenetic relationships within *Laspionchis* based on concatenated nuclear ITS2 and 28S DNA sequences for 41 individuals (including 19 outgroups). Numbers by the branches are the bootstrap values (Maximum Likelihood analysis, ML) and the posterior probabilities (Bayesian analysis, B). Only numbers > 60% (ML) and > 0.9 (B) are indicated. Numbers for each individual correspond to unique identifiers for DNA extraction. All sequences of *Laspionchis* individuals are new. Outgroups sequences are from our previous studies (Dayrat et al. 2011, 2016, 2017, 2018, 2019; Dayrat and Goulding 2017; Goulding et al. 2018a, b, c). Information on specimens can be found in the lists of material examined and in Table 1. The color used for each subspecies is the same as the color used in Figs 2, 3, 5–7.

**Figure 5.**
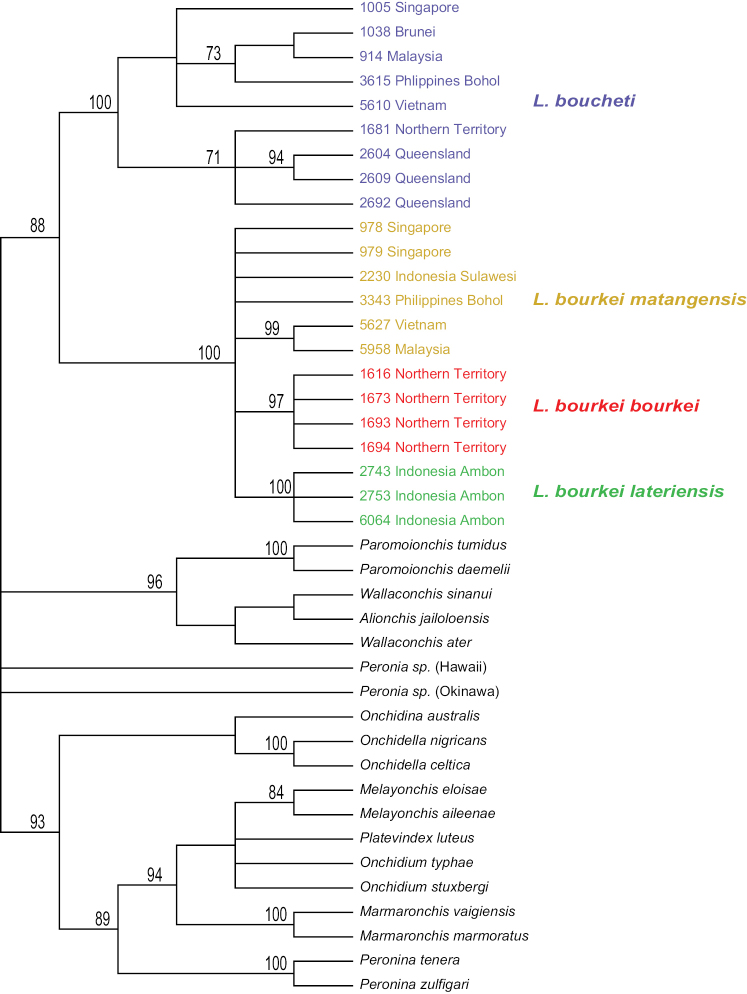
Maximum parsimony consensus tree within *Laspionchis*, performed with concatenated nuclear ITS2 and 28S DNA sequences from 41 individuals (including 19 outgroups). Numbers by the branches are the bootstrap values (only numbers > 70% are indicated). Numbers for each individual correspond to unique identifiers for DNA extraction. All sequences of *Laspionchis* individuals are new. Outgroups sequences are from our previous studies (Dayrat et al. 2011, 2016, 2017, 2018, 2019; Dayrat and Goulding 2017; Goulding et al. 2018a, b, c). Information on specimens can be found in the lists of material examined and in Table 1. The color used for each subspecies is the same as the color used in Figs 2–4, 6, 7.

DNA sequences were used to test species limits within *Laspionchis*. The monophyly of *Laspionchis* is strongly supported in all analyses (Figs 2–5). In the analyses based on mitochondrial COI and 16S concatenated sequences, there are four least-inclusive units that are all reciprocally monophyletic: *L. boucheti*, *L. bourkei bourkei*, *L. bourkei lateriensis*, and *L. bourkei matangensis* (Fig. 2). The monophyly of each unit is strongly supported by a bootstrap support of 100 and a posterior probability of 1. The monophyly of *L. bourkei* is also strongly supported (bootstrap support of 99 and a posterior probability of 1). Analyses with nuclear sequences (ITS2 alone, and concatenated ITS2 and 28S) yielded results similar to the mitochondrial sequences (Figs 3–5). *Laspionchis boucheti*, *L. bourkei*, *L. bourkei bourkei*, and *L. bourkei lateriensis* are strongly supported in all nuclear analyses: the relationships between individuals of *Laspionchis bourkei matangensis* are unresolved in nuclear analyses.

### Pairwise genetic divergences

**Figure 6.**
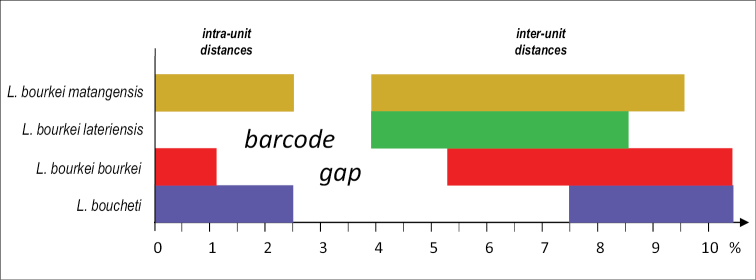
Diagram to help visualize the data on pairwise genetic distances between COI sequences within and between mitochondrial units in *Laspionchis* (see Table 3). Ranges of minimum to maximum distances are indicated (in percentages). For instance, within *L. boucheti*, individual sequences are between 0 and 2.5% divergent; individual sequences between *L. boucheti* and the other units are minimally 7.5% and maximally 10.4% divergent; overall, the distance gap between *L. boucheti* and *L. bourkei* is of 5% (i.e., between 2.5% and 7.5%). The colors are the same as those used in Figs 2–5, 7.

Pairwise genetic distances (between COI sequences) support the existence of four least-inclusive molecular units of *Laspionchis* (Table 2, Fig. 6). The intra-unit genetic distances are all below 2.5%: below 2.5% within *L. boucheti*, below 2.5% within *L. bourkei matangensis*, below 1.2% within *L. bourkei lateriensis*, and 0% within *L. bourkei lateriensis*. The inter-unit distances vary from 3.9% (the distance between *L. bourkei lateriensis* and *L. bourkei matangensis*) to 10.4% (the distance between *L. boucheti* and *L. bourkei bourkei*). So, overall, the distance gap between *L. boucheti* and *L. bourkei* is between 2.5% and 7.5%, and the distance gap between the three *L. bourkei* units (*L. bourkei bourkei*, *L. bourkei lateriensis*, and *L. bourkei matangensis*) is between 2.5% and 3.9%.

**Table 2.** Intra- and inter-unit pairwise genetic distances between the four mitochondrial units of *Laspionchis* based on our data set of 61 COI sequences (Table 1). Ranges of minimum to maximum distances are indicated (as percentages): e.g., individual sequences within *L. boucheti* are between 0 and 2.7% divergent, and individual sequences between *L. boucheti* and *L. bourkei bourkei* are minimally 8.6% and maximally 10.4% divergent. Overall, the distance gap between all four mitochondrial units is between 2.5% (the maximum intra-unit distance within *L. boucheti* and within *L. bourkei matangensis*) and 3.9% (the minimum distance between *L. bourkei lateriensis* and *L. bourkei matangensis*). Finally, the distance gap between the two species *L. boucheti* and *L. bourkei* is between 2.5% and 7.5%.

Species	*L. boucheti*	*L. bourkei*		
		* bourkei *	* lateriensis *	* matangensis *
*L. boucheti*	0.0–2.5			
*L. bourkei bourkei*	8.6–10.4	0.0–1.2		
*L. bourkei lateriensis*	7.5–8.5	6.1–7.8	0.0–0.0	
*L. bourkei matangensis*	7.5–9.5	5.3–6.1	3.9–5.5	0.0–2.5

### Comparative anatomy

In the field, slugs were numbered individually without being assigned to any particular species because onchidiid species are commonly cryptic externally. As anticipated, *Laspionchis boucheti* and *L. bourkei* are externally cryptic (Table 3). However, *Laspionchis boucheti* differs from *L. bourkei* in internal anatomy, and they cannot be confused: in *L. boucheti*, the long retractor muscle of the penis inserts at the posterior end of the visceral cavity, while the retractor muscle is absent, vestigial, or short (inserting in the first third of the visceral cavity) in *L. bourkei*. Also, additional, distal, retractor muscle fibers are present in *L. boucheti* but absent in *L. bourkei*. However, the three subspecies of *L. bourkei* are hardly distinguishable anatomically (Table 3).

**Table 3.** Summary of traits that can help distinguish the two species of *Laspionchis*. All traits are subject to individual variation. Traits are described in detail in the corresponding species descriptions. Traits are also indicated for the three subspecies of *L. bourkei*.

Species	Retractor muscle (penis)	Retractor muscle (penis) attachment site	Distal muscle fibers	Accessory penial gland spine size (mm)	Penial hooks (μm)	Distribution
*L. boucheti*	Strong and long	Posterior end of visceral cavity (by the rectum)	yes	0.7 to 1	60 to 160	Peninsular Malaysia, Indonesia (Sumatra), Singapore, Brunei, Vietnam, Philippines (Bohol), Australia (Northern Territory, Queensland)
*L. bourkei bourkei*	Very short	Anterior third of the visceral cavity	no	0.75 to 1	20 to 35	Australia (Northern Territory)
*L. bourkei lateriensis*	Absent or vestigial	–	no	0.35 to 0.75	20 to 45	Indonesia (Ambon)
*L. bourkei matangensis*	Absent or vestigial	–	no	0.43 to 0.57	15 to 40	Peninsular Malaysia, Indonesia (Sulawesi, Sumatra), Singapore, Philippines (Bohol), Vietnam

### Species delineation

The new genus described here, *Laspionchis*, is a strongly-supported clade in all molecular analyses (Figs 2–5). It also is characterized by a unique combination of anatomical characters (see below, the Remarks on the genus diagnosis). Two species are recognized here, *Laspionchis boucheti* and *L. bourkei*, which are cryptic externally but distinct anatomically (Table 3). Their reciprocal monophyly is strongly supported by both nuclear and mitochondrial sequences (Figs 2–5) and they are separated by a clear barcode gap (from 2.5% to 7.5%) in genetic distances between COI sequences (Table 2, Fig. 6). In addition, three subspecies are recognized within *Laspionchis bourkei*: *L. bourkei bourkei*, *L. bourkei lateriensis*, and *L. bourkei matangensis*, which are cryptic externally and hardly distinguishable internally (Table 3). Their reciprocal monophyly is strongly supported by mitochondrial sequences as well as by nuclear sequences (Figs 2–5) even though *L. bourkei matangensis* is unresolved in nuclear analyses. All three subspecies of *L. bourkei* are separated by a barcode gap in genetic distances between COI sequences (from 2.5% to 3.9%) which, as expected, is not as large as the gap found between *L. boucheti* and *L. bourkei* (Table 2, Fig. 6). The ranking of the three least-inclusive units within *L. bourkei* as subspecies is discussed in the general discussion.

### Systematics and anatomical descriptions

#### Family Onchidiidae Rafinesque, 1815

##### Genus Laspionchis

Taxon classificationAnimaliaSystellommatophoraOnchidiidae

Dayrat & Goulding gen. nov.

D819240C-8F69-5F69-953D-8549D3F5CC14

http://zoobank.org/47CA237B-3E0F-49BF-A866-C551E979A236

###### Type species.

*Laspionchis boucheti*, designated here.

###### Etymology.

Combination of *láspi*, a Greek word meaning mud, and *onchis*, a word derived from the Greek *ὁ ὂγκος* (mass, tumor) and used in the past for onchidiid slugs. *Laspionchis* conveniently refers to those onchidiid species that always live on mud and are covered with a thin layer of mud.

###### Gender.

Gender masculine of *onchis* (ICZN Art. 30.1.1), a word derived from the masculine Greek word *ὁ ὂγκος*.

###### Diagnosis.

Body not flattened. No dorsal gills. Dorsal eyes present on notum. Retractable, central papilla (usually with four dorsal eyes) present, often raised above dorsal surface. Eyes at tip of short ocular tentacles. Male opening below right ocular tentacle (or below it and very slightly to its left). No transversal protuberance on oral lobes. Foot wide. Pneumostome median, on ventral hyponotum. Intestinal loops exactly between types I and II (with a transitional loop on average descending at 6 o’clock). Rectal gland absent. Accessory penial gland present with a hollow spine and a muscular sac. Penis with hooks: numerous, densely arranged next to each other, and pointed.

###### Remarks.

No external diagnostic feature unambiguously distinguishes *Laspionchis* from other onchidiid genera. Externally, *Laspionchis* slugs are especially difficult to distinguish from *Paromoionchis* slugs, which live in the same habitat (mud surface) and are often found together at the exact same sites. Also, for a non-expert, *Laspionchis* slugs could easily be confused with *Peronina* or *Onchidium* slugs, although those are characterized by distinctive, external features. However, *Laspionchis* is characterized by a unique combination of internal and external characters: no dorsal gills, male opening below the right eye tentacle (or below it and very slightly to its left), no rectal gland, intestinal loops between types I and II (i.e., with a transitional loop on average oriented at 6 o’clock), accessory penial gland present with a muscular sac, penis with numerous, pointed hooks densely arranged next to each other. According to our data, any onchidiid slug with this combination of characters belongs to *Laspionchis*.

Intestinal loops between types I and II, with a transitional loop on average oriented at 6 o’clock, could almost be regarded as diagnostic of *Laspionchis* slugs, acknowledging the existence of variation (both intra-specific and inter-specific). Indeed, in *Laspionchis* slugs, the transitional loop is normally oriented at 6 o’clock, even though, strictly speaking, its orientation actually varies between 5 and 7 o’clock (Fig. 1). The intestinal loops of some individuals of other species can sometimes be characterized by a transitional loop oriented within that same range (between 5 and 7 o’clock), such as in species with intestinal loops of type I and a transitional loop oriented from 3 to 6 o’clock (as in *Wallaconchis*, see Goulding et al. 2018a), and in species with intestinal loops of type II and a transitional loop oriented from 6 to 9 o’clock (as in *Paromoionchis*, see Dayrat et al. 2019). However, the important difference here is that a transitional loop oriented at 6 o’clock is the norm in *Laspionchis*, while it is not the norm in those species from other genera.

A new generic name is needed because no existing name applies to the clade described here. Based on the examination of all the type specimens available in Onchidiidae (especially those of all the type species), a careful study of all the original descriptions (especially when no type specimens were available), and our ongoing taxonomic revision of every genus of the family (Dayrat et al. 2016, 2017, 2018, 2019; Dayrat and Goulding 2017; Goulding et al. 2018a, b, c), it appears that there is no generic name of which the type species matches the diagnosis of this genus. For a recent review of the application of all existing generic names of Onchidiidae, see Dayrat et al. (2017: 1861).

##### Laspionchis boucheti

Taxon classificationAnimaliaSystellommatophoraOnchidiidae

Dayrat & Goulding sp. nov.

EE64725D-E976-5C56-8DE1-720DD37A5033

http://zoobank.org/41EC4683-63DD-4C15-B3CA-F0FB6E543A33

Figs 8 , 9 , 10 , 11 , 12 , 13 , 14 , 15

###### Holotype.

AUSTRALIA • holotype, designated here, 30/20 mm [1688 H]; Northern Territory, Darwin, end of the Channel Island Road; 12°33.557'S, 130°52.894'E; 17 Aug. 2012; B Dayrat and party leg.; st 66, sequence of *Sonneratia*, *Rhizophora*, and *Ceriops*; NTM P.57614.

###### Additional material examined.

AUSTRALIA – **Northern Territory** • 2 specimens 25/20 mm [1679], 35/25 mm [1681]; Darwin, near Channel Island Road; 12°34.979'S, 130°55.992'E; 16 Aug. 2012; B Dayrat and party leg.; st 65, sequence of *Sonneratia*, *Rhizophora*, and *Ceriops*; NTM P.57612. • 1 specimen 16/10 mm [1685]; same collection data as for the holotype; NTM P.57613. – **Queensland** • 1 specimen 15/10 mm [2559]; Cairns, Yorkey’s Knob; 16°48.558'S, 145°42.768'E; 17 Jun. 2013; TC Goulding and party leg.; st 101, hard, red mud with grasses; MTQ. • 1 specimen 15/9 mm [2578]; Cairns, Yorkey’s Knob; 16°48.503'S, 145°42.869'E; 21 Jun. 2013; TC Goulding and party leg.; st 105, hard, red mud with grasses; MTQ. • 1 specimen 18/15 mm [2593]; Townsville, Magnetic Island; 19°09.733'S, 146°48.625'E; 24 Jun. 2013; TC Goulding and party leg.; st 108, very soft mud near creek, with some *Rhizophora* and *Avicennia* trees on sides; MTQ. • 1 specimen 16/15 mm [2604]; Townsville; 19°17.717'S, 146°49.487'E; 25 Jun. 2013; TC Goulding and party leg.; st 110, mangrove of short and dense trees; MTQ. • 2 specimens 21/14 mm [2609], 30/17 mm [2612]; Townsville, Ross River; 19°16.275'S, 146°50.284'E; 26 Jun. 2013; TC Goulding and party leg.; st 111, open forest of young *Avicennia*, very soft mud; MTQ. • 1 specimen 26/20 mm [2692], 30/17 mm [2693]; Mackay, Barnes Creek; 21°07.815'S, 149°11.396'E; 7 Jul. 2013; TC Goulding and party leg.; st 124, soft mud, open area with *Avicennia* and some Rhizophora, wetland restoration area; MTQ. BRUNEI DARUSSALAM • 2 specimens 31/20 mm [1037], and 14/8 mm [1038]; Pulau Pyatan, Teluk Brunei; 04°55.246'N, 115°02.764'E; 27 Jul. 2011; B Dayrat and party leg.; st 32, open mangrove with a few sparse old trees, and large old logs, by the river; BDMNH. INDONESIA – **Sumatra** • 1 specimen 15/10 mm [1729]; Tembilahan; 00°10.243'S, 103°27.982'E; 13 Oct. 2012; M Khalil and party leg.; st 76, mangrove of large *Avicennia* trees, with old logs, soft but solid mud, and *Nypa* on the margin; UMIZ 00112. MALAYSIA – **Peninsular Malaysia** • 1 specimen 20/10 mm [914]; Matang, facing fishermen’s village on the other side of river; 04°50.154'N, 100°36.368'E; 20 Jul. 2011; B Dayrat and party leg.; st 29, oldest and open *Rhizophora* forest of tall and beautiful trees, with hard mud, many creeks, and many old logs; USMMC 00054. • 1 specimen 12/8 mm [915]; Matang, off Kuala Sepatang, Crocodile River, Sungai Babi Manpus; 04°49.097'N, 100°37.370'E; 19 Jul. 2011; B Dayrat and party leg.; st 28, old and open *Rhizophora* forest with tall trees, hard mud, creeks, and many old logs; USMMC 00053. • 1 specimen 10/8 mm [5520]; Kuala Sepatang; 04°50.434'N, 100°38.176'E; 19 Jul. 2011; B Dayrat and party leg.; st 27, old forest with tall, old *Rhizophora* trees, high in the tidal zone (ferns), in educational mangrove preserve, at a creek lower in the tidal zone, with mud; USMMC 00052. PHILIPPINES – **Bohol** • 1 specimen 14/11 mm [3615]; Inabanga; 10°00.389'N, 124°03.522'E; 12 Jul. 2014; B Dayrat and party leg.; st 186, old, rehabilitated fish ponds next to a mangrove with some old *Avicennia* but mostly young *Rhizophora* trees; PNM 041252. SINGAPORE • 1 specimen 15/12 mm [1004]; Lim Chu Kang, 01°26.785'N, 103°42.531'E; 5 Apr. 2010; B Dayrat and party leg.; st 9, east of the jetty, open mangrove with medium trees, ending on mudflat outside mangrove with soft mud; ZRC.MOL.10483. • 1 specimen 10/8 mm [1005]; Pasir Ris Park; 01°22.840'N, 103°57.224'E; 30 Mar. 2010; B Dayrat and party leg.; st 5, mangrove forest with rich litter, lobster mounds, and old logs; ZRC.MOL.10482. VIETNAM • 2 specimens 31/20 mm [5609], 39/25 mm [5610]; Can Gio; 10°27.620'N, 106°53.316'E; 17 Jul. 2015; TC Goulding and party leg.; st 231, open mangrove with large *Avicennia* trees, soft mud, some old logs; ITBZC IM 00017.

###### Distribution

**Figure 7.**
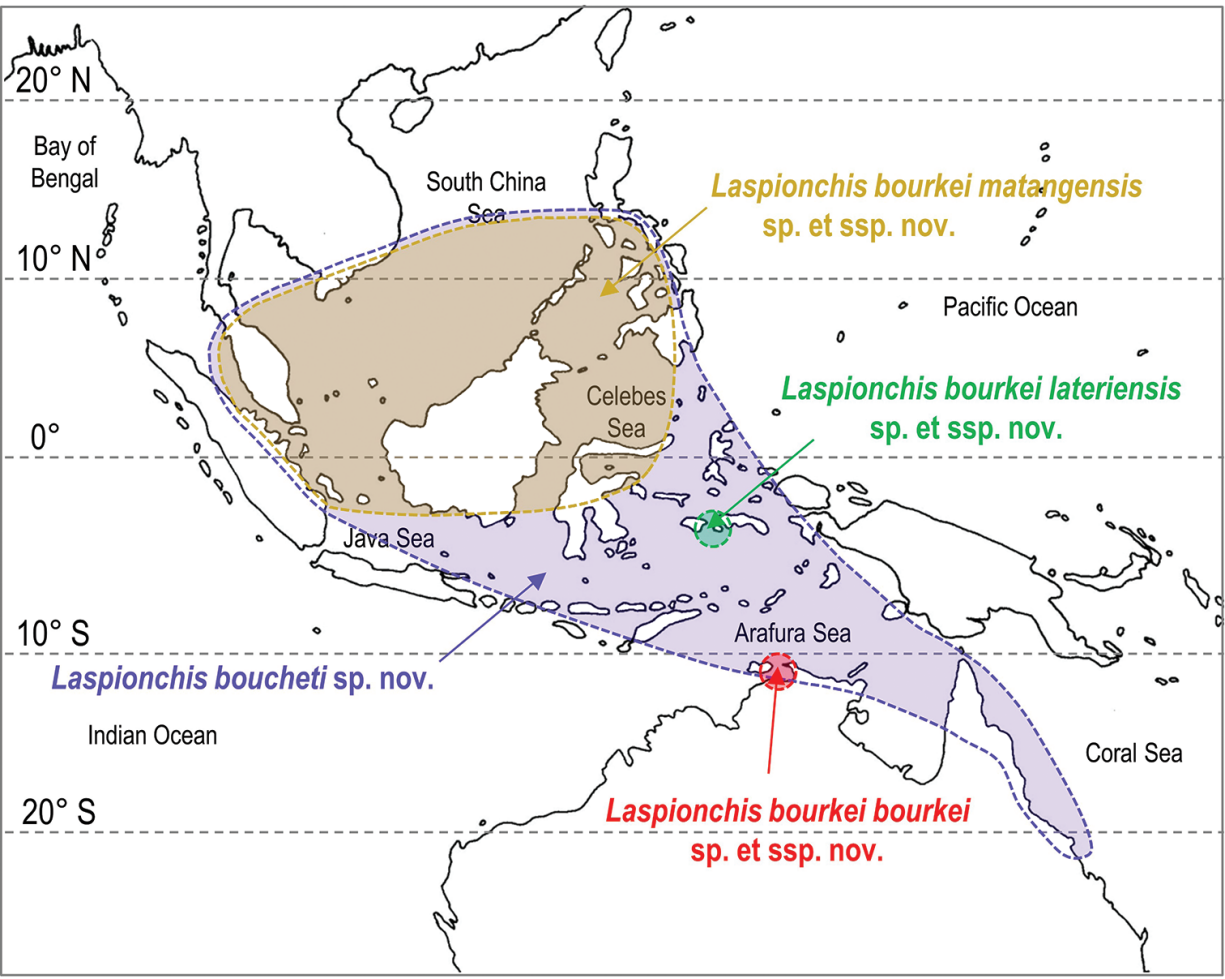
Geographical distribution of the two species of *Laspionchis*. Distinct colors are used for each subspecies of *L. bourkei*. The colors used are the same as those used in the phylogenetic trees (Figs 2–5). Colored areas correspond to hypothetical geographical ranges based on known records.

(Fig. 7). Australia: Northern Territory, Queensland. Brunei Darussalam. Indonesia: Sumatra. Malaysia: Peninsular Malaysia. Singapore. Philippines: Bohol. Vietnam.

###### Habitat

**Figure 8.**
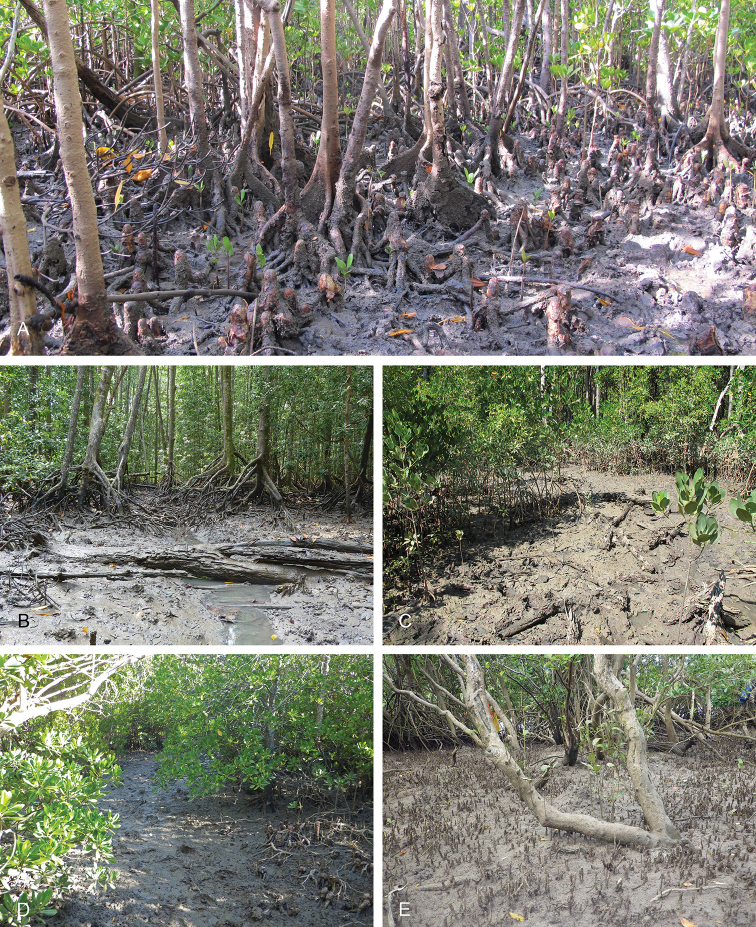
Habitats, *Laspionchis boucheti*. **A** Australia, Northern Territory, *Sonneratia*, *Rhizophora*, and *Ceriops* mangrove (st 66, type locality) **B** Peninsular Malaysia, *Rhizophora*, hard mud, open space, old forest (st 29) **C** Australia, Northern Territory, *Sonneratia*, *Rhizophora*, and *Ceriops* mangrove (st 65) **D** Australia, Queensland, mangrove of short shrubs and dense trees (st 110) **E** Australia, Queensland, soft mud, open area with *Avicennia*, some *Rhizophora* (st 124).

(Fig. 8). *Laspionchis boucheti* is found on mud, hard or soft, in open or dense mangrove forests. It is common across its entire distribution range.

###### Etymology.

*Laspionchis boucheti* is dedicated to Philippe Bouchet, professor of Malacology at the Muséum national d’Histoire naturelle, Paris, France, for the training that he generously provided to the first author as a graduate student at the MNHN, years ago, for kindly allowing us to study some material collected during expeditions that he organized (Kavieng, Madagascar, New Caledonia, Papua New Guinea, Vanuatu), and, more broadly, for his unconditional love of snails and slugs, biodiversity exploration, and alpha-taxonomy.

###### Diagnosis

(Table 3). Externally, *Laspionchis boucheti* cannot be distinguished from *L. bourkei*. Internally, however, the insertion of the retractor muscle of the penis (at the posterior end of the visceral cavity) and the presence of additional, distal retractor muscle fibers can help distinguish *L. boucheti* from *L. bourkei*.

###### Color and morphology of live animals

**Figure 9.**
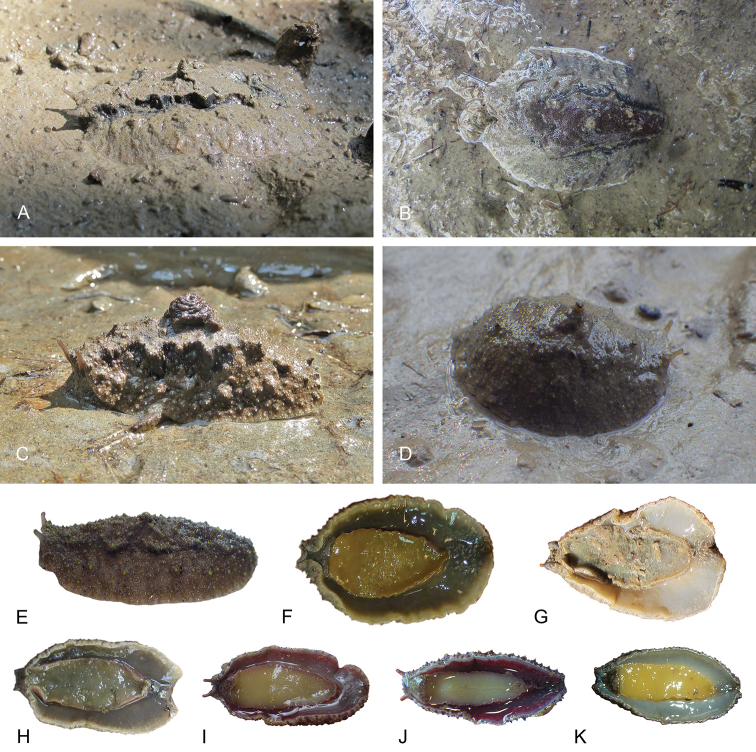
Live animals, *Laspionchis boucheti*. **A** Dorsal view, 35 mm long [1681], Australia, Northern Territory (NTM P.57612) **B** holotype, dorsal view, 30 mm long [1688 H], Australia, Northern Territory (NTM P.57614) **C** dorsal view, 31 mm long [1037], Brunei (BDMNH) **D** dorsal view, 15 mm long [1729], Indonesia, Sumatra (UMIZ 00112) **E** dorsal view, 30 mm long [2612], Australia, Queensland (MTQ) **F** ventral view, same as **C G** ventral view, same as **A H** ventral view, same as **B I** ventral view, 21 mm long [2609], Australia, Queensland (MTQ) **J** ventral view, 26 mm long [2692], Australia, Queensland (MTQ) **K** ventral view, 20 mm long [914], Peninsular Malaysia (USMMC 00054).

**Figure 10.**
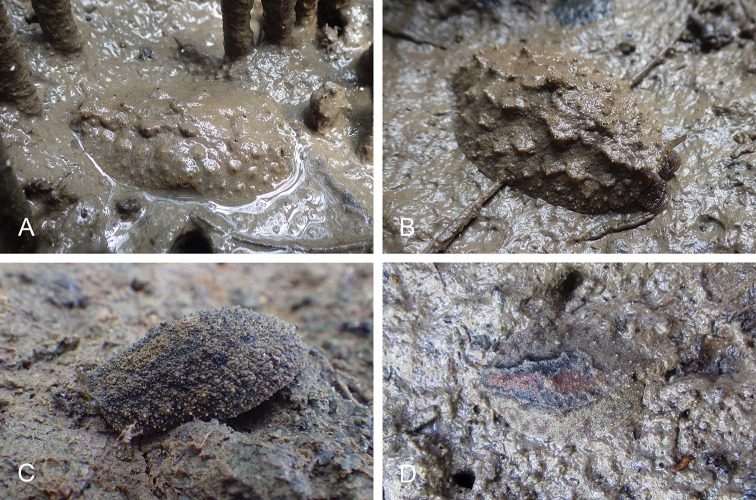
Live animals, *Laspionchis boucheti*, dorsal view. **A** 39 mm long [5610], Vietnam, Can Gio (ITBZC IM 00017) **B** 31 mm long [5609], Vietnam, Can Gio (ITBZC IM 00017) **C** 15 mm long [2578], Australia, Queensland (MTQ) **D** 16 mm long [1685], Australia, Northern Territory (NTM P.57613).

(Figs 9, 10). Live slugs are covered with mud and their dorsal color can hardly be seen. The background of the dorsal notum is brown, light to dark, homogenous or mottled with darker or lighter areas, and, occasionally, with red areas too. In some slugs, the tip of dorsal papillae (with and without dorsal eyes) can be yellow. The color of the foot is gray (light or dark), yellow, or orange. The hyponotum is light or dark grey, pale yellow or red, sometimes with a lighter whitish margin. The color of the foot and of the hyponotum of an individual can change rapidly, especially when disturbed. The ocular tentacles are brown (variable from light to dark) and short (a few millimeters).

Generally, the dorsal notum of any given slug can rapidly change from almost perfectly smooth to densely covered by many papillae. However, when slugs are not disturbed, the dorsum is usually covered by papillae of various sizes. In some slugs, larger papillae may be arranged in two longitudinal ridges on either side of the median line, but those ridges can appear and disappear rapidly. Some papillae bear dorsal eyes at their tip (most papillae bear three eyes). The number of papillae with dorsal eyes is variable (between 8 and 12, on average) and they mostly are on the central part of the notum. Their tip can be pale yellow, but not always. A central, much larger papilla, which also bears three dorsal eyes, is entirely retractable within the notum. In addition to the large papillae, the notum is covered by smaller, rounded papillae, which can make it look very granular.

###### External morphology

**Figure 11.**
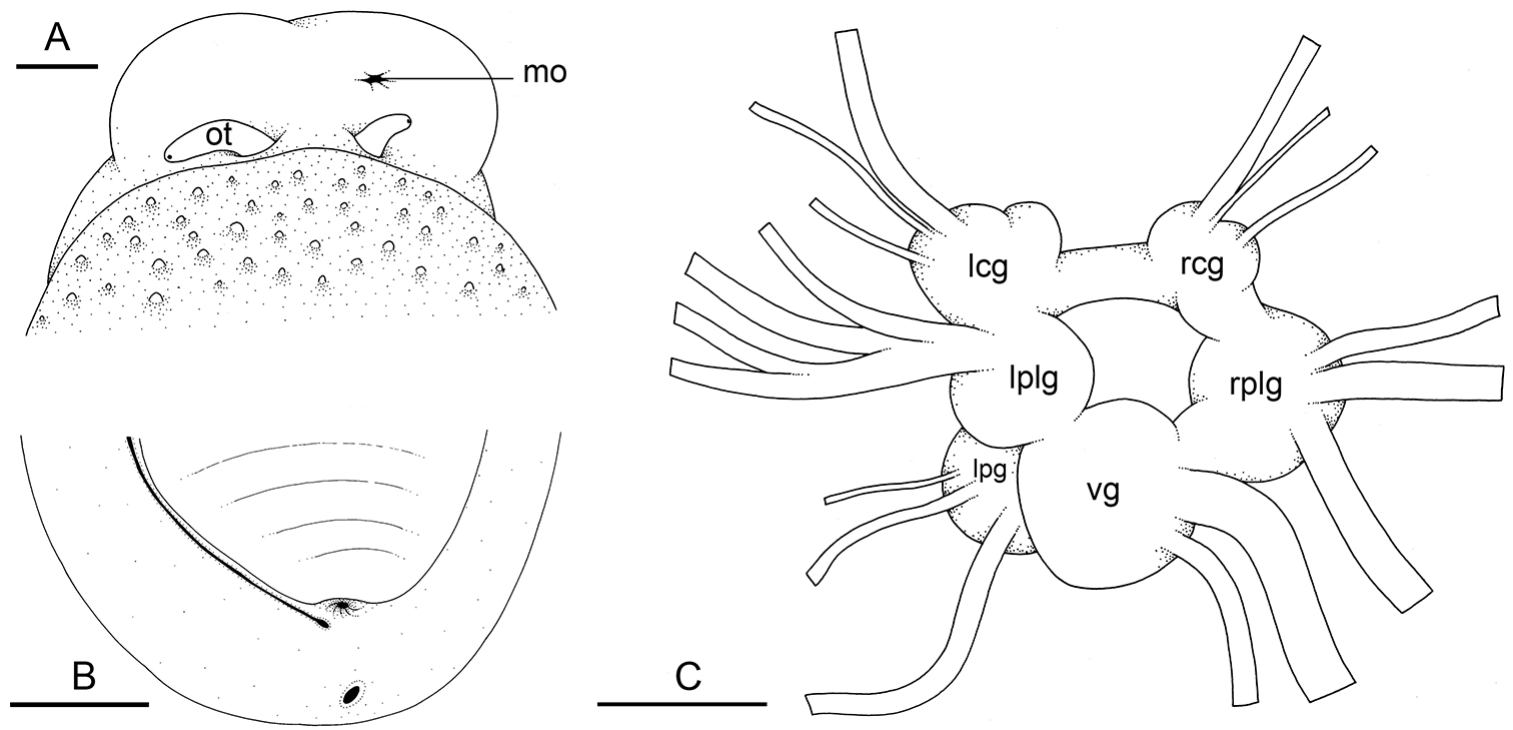
External morphology and nervous system, *Laspionchis boucheti***A** Australia, Queensland [2693] (MTQ) **B, C** holotype, Australia, Northern Territory [1688 H] (NTM P.57614). **A** Dorsal, anterior view **B** ventral, posterior view **C** nervous system, dorsal view. Scale bars: 2 mm (**A**), 3 mm (**B**), 0.5 mm (**C)**. Abbreviations: lcg left cerebral ganglion lpg left pedal ganglion lplg left pleural ganglion mo male opening ot ocular tentacle rcg right cerebral ganglion rplg right pleural ganglion vg visceral ganglion.

(Fig. 11A, B). The body is not flattened. The notum is oval. Dorsal gills are absent. The large, central, retractable papilla at the center of the notum can usually only be seen in live animals. In preserved specimens, it is retracted inside the notum. The hyponotum is horizontal. The width of the hyponotum relative to the width of the pedal sole varies among individuals. The width of the hyponotum is approximately half of its total width. In the anterior region, the left and right ocular tentacles are superior to the mouth. Eyes are located at the tip of the ocular tentacles. Inferior to the ocular tentacles, superior to the mouth, the head bears a pair of oral lobes. The latter are smooth, with no transversal protuberance. The male aperture (opening of the copulatory complex) is below the right ocular tentacle (or very slightly to its left in dorsal view). The anus is posterior, medial, close to the edge of the pedal sole. On the right side (to the left in ventral view), a peripodial groove is present at the junction between the pedal sole and the hyponotum, running longitudinally from the buccal area to the posterior end, very close to the anus. The position of the female pore (at the posterior end of the peripodial groove) does not vary much among individuals. The pneumostome is medial. Its position on the hyponotum relative to the notum margin and the edge of the pedal sole varies among individuals but it tends to be closer to the notum margin.

###### Visceral cavity and pallial complex.

The heart, enclosed in the pericardium, is on the right side of the visceral cavity, slightly posterior to the middle. From the anterior ventricle is an anterior vessel supporting several anterior organs such as the buccal mass, the nervous system, and the copulatory complex. The auricle is posterior. The kidney is more or less symmetrical, the right and left parts being equally developed. The kidney is intricately attached to the respiratory complex. The lung is in two left and right, more or less symmetrical, parts.

###### Digestive system

**Figure 12.**
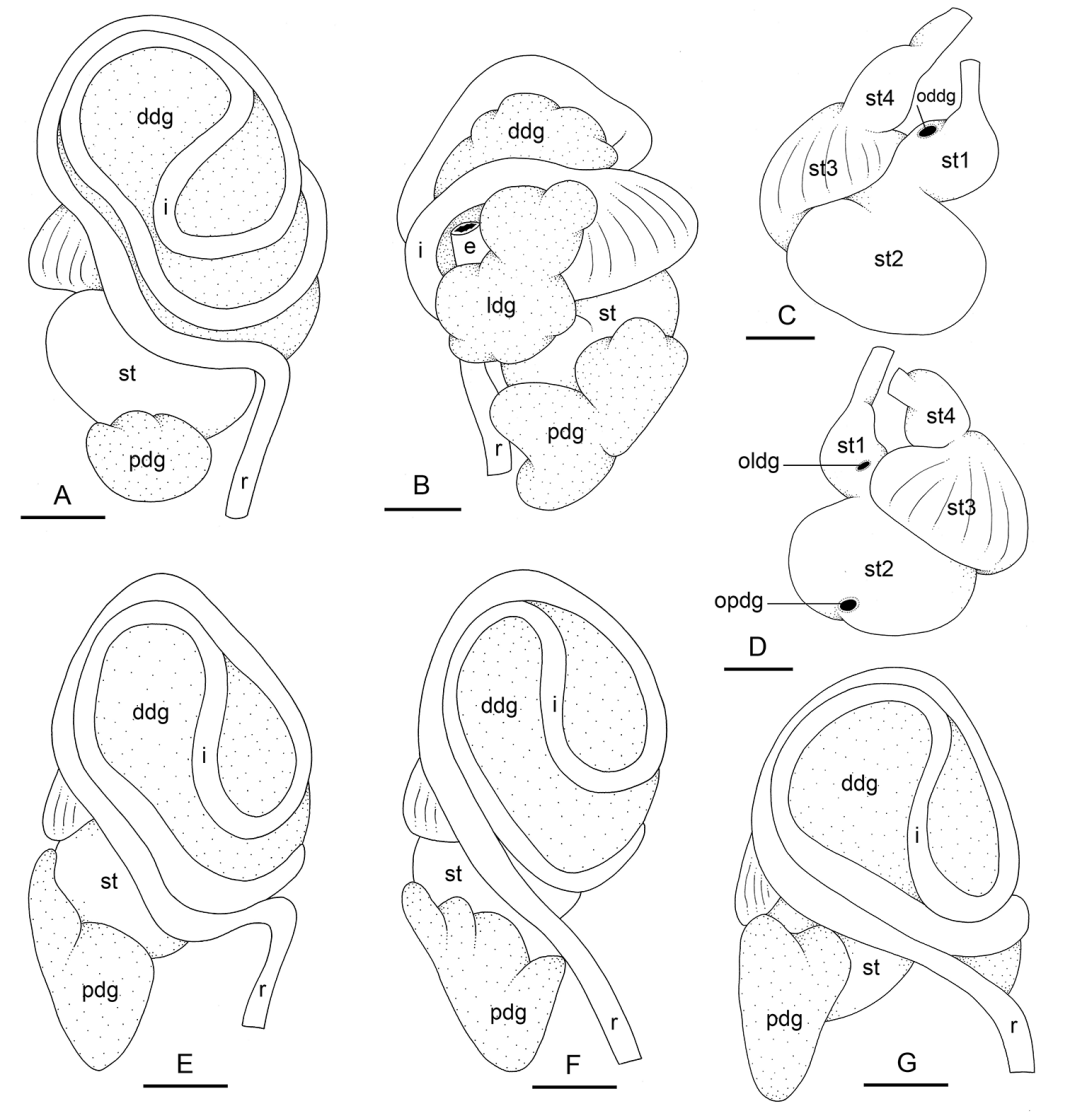
Digestive system, *Laspionchis boucheti*. **A** Holotype, dorsal view, Australia, Northern Territory, [1688 H] (NTM P.57614) **B** ventral view, same as **A C** stomach, dorsal view, same as **A D** stomach, ventral view, same as **A E** dorsal view, Vietnam, [5610] (ITBZC IM 00017) **F** dorsal view, Australia, Queensland, [2612] (MTQ) **G** dorsal view, Australia, Northern Territory, [1681] (NTM P.57612). Scale bars: 2 mm (**A–D**) 3 mm (**E, G)** 2.5 mm (**F**). Abbreviations: ddg dorsal digestive gland e esophagus i intestine ldg lateral digestive gland mo male opening oddg opening of the dorsal digestive gland oldg opening of the lateral digestive gland opdg opening of the posterior digestive gland pdg posterior digestive gland r rectum st stomach st1 stomach chamber 1 st2 stomach chamber 2 st3 stomach chamber 3 st4 stomach chamber 4.

**Figure 13.**
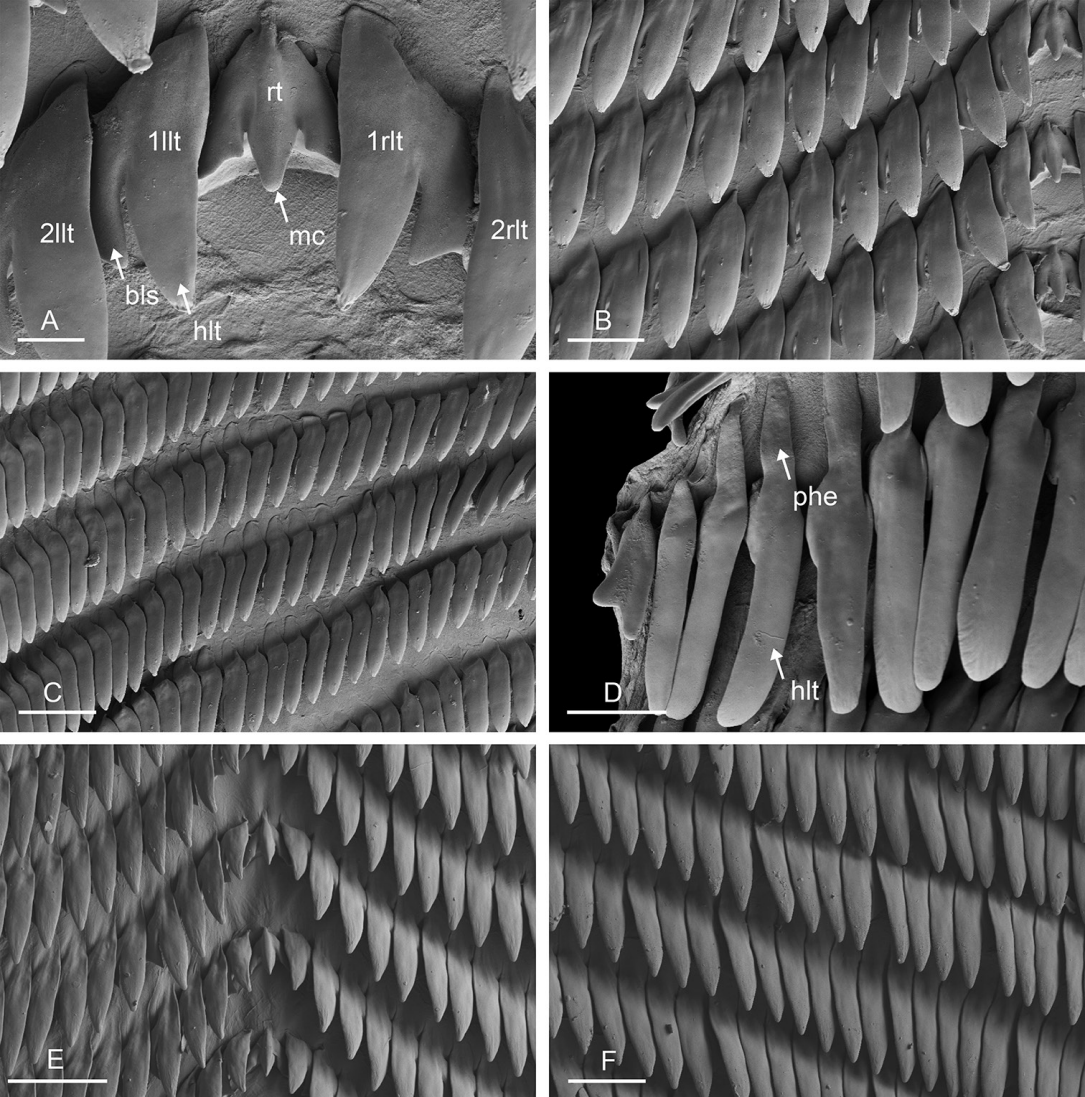
Radula, *Laspionchis boucheti***A–D** Vietnam [5609] (ITBZC IM 00017) **E, F** holotype, Australia, Northern Territory [1688 H] (NTM P.57614). **A** Rachidian and innermost lateral teeth **B** lateral teeth with rachidian teeth **C** lateral teeth **D** outermost lateral teeth **E** rachidian and lateral teeth **F** lateral teeth. Scale bars: 10 μm (**A**) 30 μm (**B, F**) 50 μm (**C**) 20 μm (**D**) 40 μm (**E)**. Abbreviations: 1llt first left lateral tooth 1rlt first right lateral tooth 2llt second left lateral tooth 2rlt second right lateral tooth bls basal lateral spine hlt hook of a lateral tooth mc median cusp phe posterior hook extension rt rachidian tooth.

(Figs 12, 13). There are no jaws. The left and right salivary glands, heavily branched, join the buccal mass dorsally, on either side of the esophagus. The radula is between two large postero-lateral muscular masses. Radulae measure up to 2 mm in length. Each radular row contains a rachidian tooth and two half rows of lateral teeth of similar size and shape. Examples of radular formulae are in Table 4. The rachidian teeth are unicuspid: the median cusp is always present; there are two inconspicuous lateral cusps (Fig. 13A). The length of the rachidian teeth (ca. 20 µm) tend to be approximately half the size of the lateral teeth (ca. 50 µm). The lateral aspect of the base of the rachidian teeth is straight, occasionally slightly convex. The half rows of lateral teeth form an angle of 45° with the rachidian axis. With the exception of the few innermost and outermost lateral teeth, the size and shape of the hook of the lateral teeth do not vary along the half row, nor do they vary among half rows. The hook of lateral teeth is extended posteriorly by a tail-like structure attaching to the radular membrane and making the hook look longer. The tail-like structure (posterior hook extension, Fig. 13D) is especially obvious in the outermost lateral teeth as its length gradually increases along each half row. The lateral teeth seem to be unicuspid with a flattened and curved hook with a rounded or pointed tip, but there also is a pointed spine on the outer lateral expansion of the base (basal lateral spine, Fig. 13A). In most cases, that spine cannot be observed because it is hidden below the hook of the next, outer lateral tooth. It can only be observed when the teeth are not too close (such as in the innermost and outermost regions) or when teeth are placed in an unusual position. The length of the spine decreases along the half row such that outermost teeth may be characterized by reduced or no lateral spine. The inner and outer lateral aspects of the hook of the lateral teeth are straight (i.e., not wavy and not with a protuberance).

**Table 4.** Radular formulae for the two species of *Laspionchis*, following the format number of rows × number of lateral teeth per left half row – 1 (rachidian tooth)– number of lateral teeth per right half row. Each DNA extraction number corresponds to one individual. The voucher catalog numbers can be shared by several individuals when collected at exactly the same locality (each individual is preserved in its own separate vial with its corresponding DNA number).

Species	Radular formula	Specimen length (mm)	Voucher	DNA extraction number
*L. boucheti*	43 × 50-1-50	30	NTM P.57614	1688 H
	57 × 90-1-90	31	BDMNH	1037
	55 × 70-1-70	31	ITBZC IM 00017	5609
*L. bourkei bourkei*	55 × 75-1-75	23	NTM P.57615	1657 H
	50 × 75-1-75	32	NTM P.57618	1666
	45 × 65-1-65	25	NTM P.57619	1692
	43 × 60-1-60	19	NTM P.57618	1673
*L. bourkei lateriensis*	45 × 65-1-65	17	UMIZ 00115	6064 H
	50 × 50-1-50	18	UMIZ 00116	6063
	45 × 60-1-60	15	UMIZ 00116	6065
	40 × 55-1-55	15	UMIZ 00116	6061
*L. bourkei matangensis*	42 × 60-1-60	15	USMMC 00055	5958 H
	55 × 70-1-70	25	PNM 041254	3343
	45 × 60-1-60	12	USMMC 00056	5960
	41 × 55-1-55	15	USMMC 00056	5959
	37 × 45-1-45	8	USMMC 00056	5965

The esophagus is narrow and straight, with thin internal folds. The esophagus enters the stomach anteriorly. Only a portion of the posterior aspect of the stomach can be seen in dorsal view because it is partly covered by the lobes of the digestive gland. The dorsal lobe is mainly on the right. The left, lateral lobe is mainly ventral. The posterior lobe covers the posterior aspect of the stomach. The stomach is a U-shaped sac divided into four chambers. The first chamber, which follows the esophagus, receives the ducts of the dorsal and lateral lobes of the digestive gland. The second chamber, posterior, receives the duct of the posterior lobe of the digestive gland. The third chamber is funnel-shaped and lined by ridges internally. The fourth chamber is continuous and externally similar to the third. The intestine is long, narrow, and the intestinal loops are exactly between types I and II, i.e., with a transitional loop on average oriented at 6 o’clock, though the orientation of the transitional loop ranges between 5 and 7 o’clock (Figs 1, 12). There is no rectal gland.

###### Nervous system

(Fig. 11C). The circum-esophageal nerve ring is post-pharyngeal and pre-esophageal. The paired cerebral ganglia are close and the cerebral commissure is short (but its length does vary among individuals). Paired pleural and pedal ganglia are also all distinct. The visceral commissure is very short and the visceral ganglion is more or less median. Cerebro-pleural and pleuro-pedal connectives are short and pleural and cerebral ganglia touch each other on either side. Nerves from the cerebral ganglia innervate the buccal area and the ocular tentacles, and, on the right side, the penial complex. Nerves from the pedal ganglia innervate the foot. Nerves from the pleural ganglia innervate the lateral and dorsal regions of the mantle. Nerves from the visceral ganglia innervate the visceral organs.

###### Reproductive system

**Figure 14.**
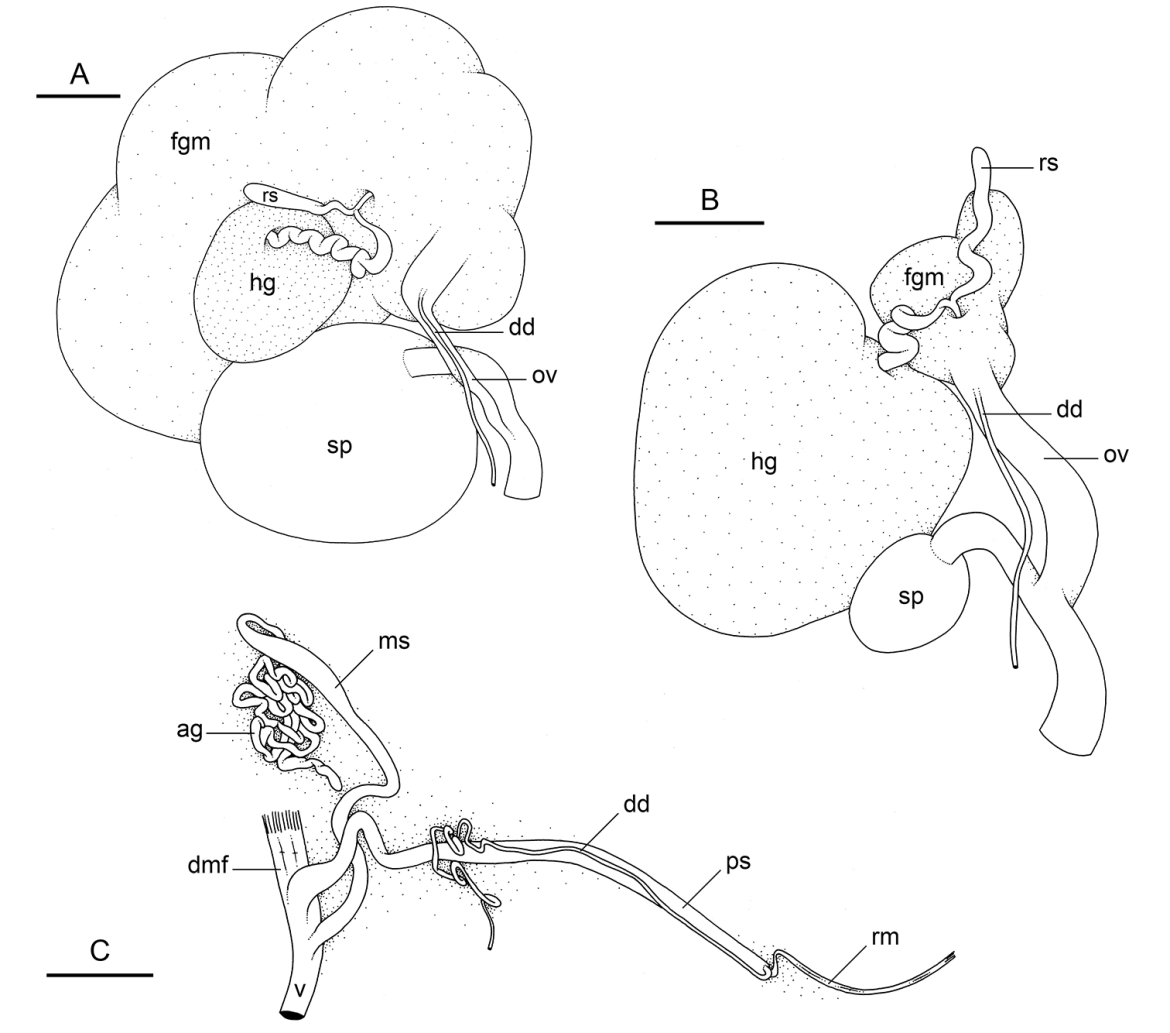
Reproductive system, *Laspionchis boucheti***A** Brunei [1037] (BDMNH) **B, C** holotype, Australia, Northern Territory [1688 H] (NTM P.57614). **A** Posterior, hermaphroditic (female) reproductive system **B** posterior, hermaphroditic (female) reproductive system **C** anterior, male, copulatory apparatus. Scale bars: 3 mm (**A**) 1 mm (**B**) 2 mm (**C)**. Abbreviations: ag accessory penial gland dd deferent duct dmf distal muscle fibers fgm female gland mass hg hermaphroditic gland ms muscular sac ov oviduct ps penial sheath rm retractor muscle rs receptaculum seminis sp spermatheca v vestibule.

(Fig. 14). Sexual maturity is correlated with animal length. Mature individuals have large female organs (with a large female gland mass) and fully-developed male parts. Immature individuals may have inconspicuous (or no) female organs and rudimentary anterior male parts. The hermaphroditic gland is a single mass, joining the spermoviduct through the hermaphroditic duct. There is a narrow receptaculum seminis (caecum) along the hermaphroditic duct. The female gland mass contains various glands (mucus and albumen) which can hardly be separated by dissection and of which the exact connections remain uncertain. The hermaphroditic duct becomes the spermoviduct. Proximally, the spermoviduct is not divided (at least externally) and is embedded within the female gland mass. Distally, the spermoviduct branches into the deferent duct and the oviduct. The free oviduct conveys the eggs up to the female opening and the exosperm from the female opening up to the fertilization chamber. The large, ovate-spherical spermatheca connects to the oviduct through a narrow and short duct. The oviduct is large (larger than the deferent duct) and straight. There is no vaginal gland.

###### Copulatory apparatus

**Figure 15.**
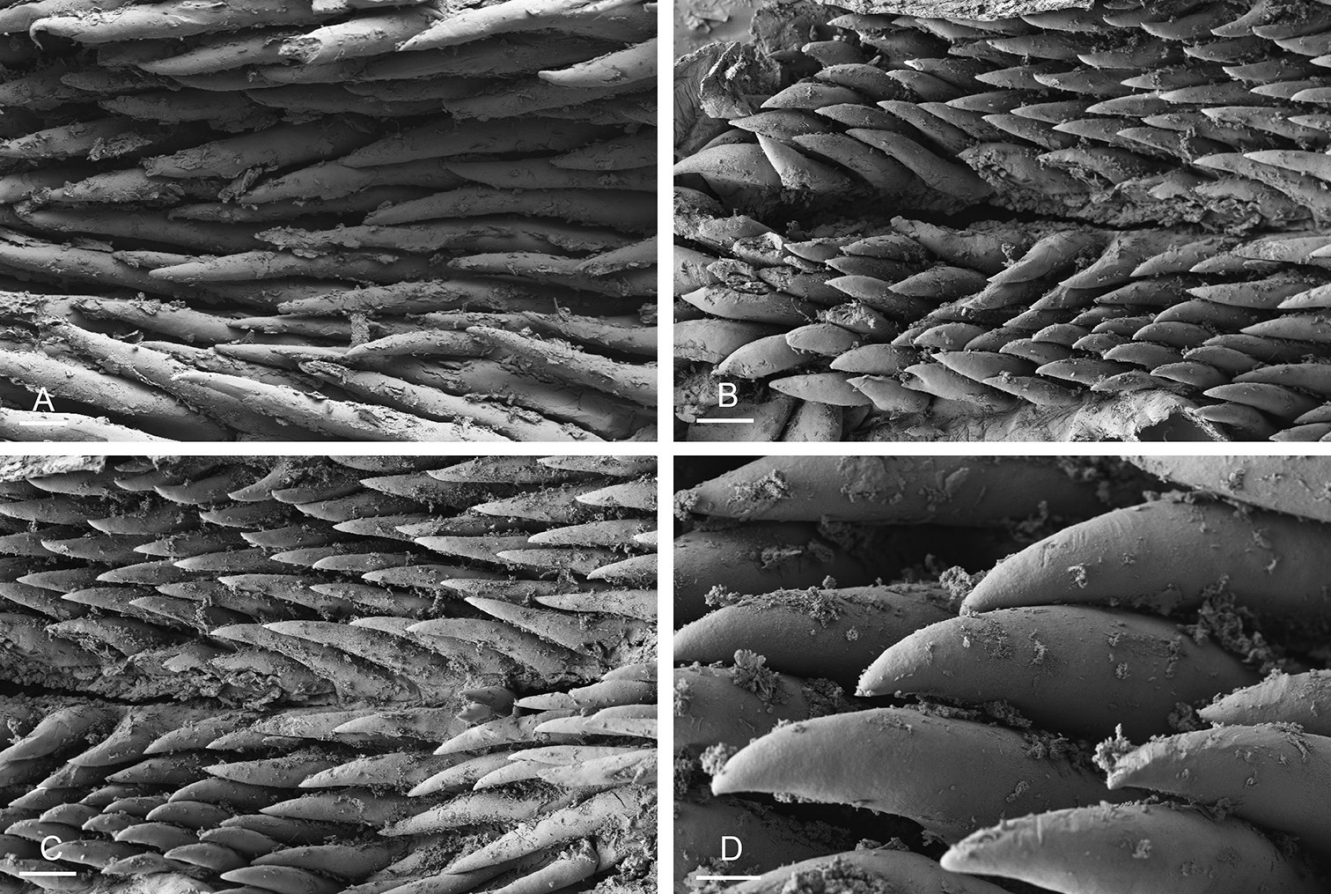
Penial hooks, *Laspionchis boucheti***A** Brunei [1037] (BDMNH) **B–D** Vietnam [5609] (ITBZC IM 00017). Scale bars: 20 μm (**A**), 40 μm (**B, C**), 10 μm (**D)**.

**Figure 16.**
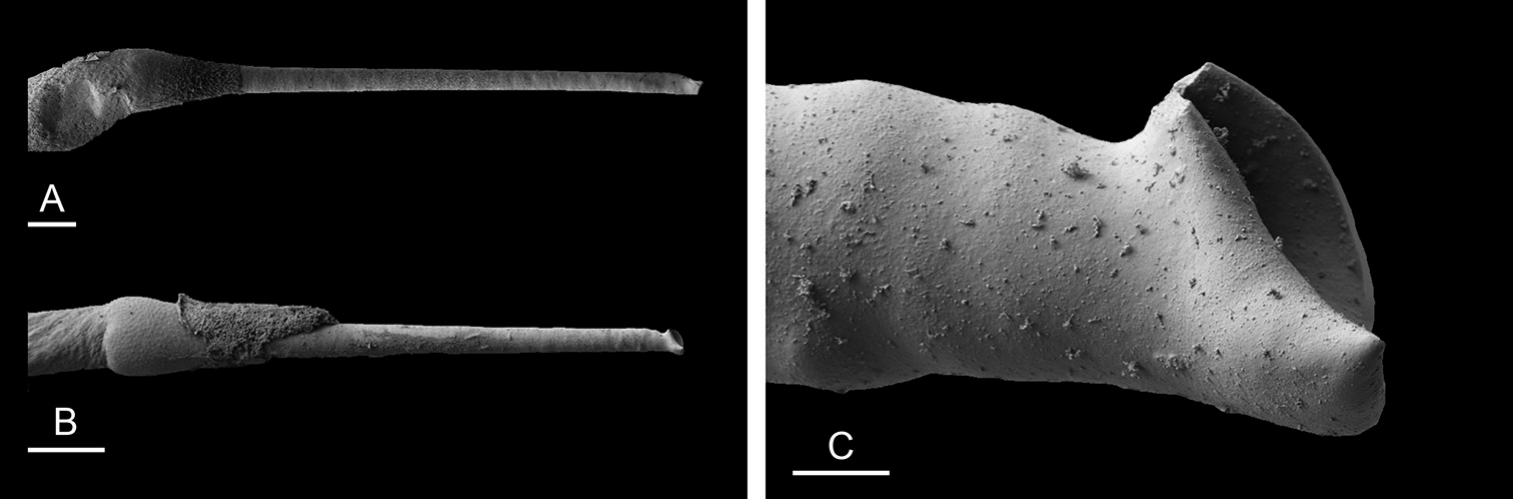
Spine of the accessory penial gland, *Laspionchis boucheti***A** Australia, Queensland [2693] (MTQ) **B, C** Brunei [1037] (BDMNH). Scale bars: 100 μm (**A, B**), 10 μm (**C)**.

(Figs 14C, 15, 16). The male anterior organs consist of the penial complex (penis, penial sheath, deferent duct, retractor muscle) and the accessory penial gland (flagellum, muscular sac, hollow spine). The penial complex and the accessory penial gland share the same vestibule and the same anterior male opening.

The penial sheath is narrow and elongated. The penial sheath protects the penis for its entire length. The beginning of the retractor muscle marks the separation between the penial sheath (and the penis inside) and the deferent duct. The retractor muscle is strong, shorter than the penial sheath, and inserts at the posterior end of the visceral cavity. In addition, there is a cluster of retractor muscle fibers on the distal part of the penial sheath, near the vestibule. The deferent duct is highly convoluted with many loops. Inside the penial sheath, the penis is a narrow, thin, elongated, hollow tube, with numerous and densely-arranged (next to each other) hooks in its distal part. Penial hooks are pointed and measure from 50 to 100 μm. When the penis is retracted inside the penial sheath, the hooks are inside the tube-like penis; during copulation, the penis is everted like a glove and the hooks are then on the outside.

The accessory penial gland is a long, tube-like flagellum with a proximal dead end. The length of the flagellum of the penial gland varies among individuals but it is always heavily coiled. Near its distal part, the flagellum is enlarged into a muscular sac. Distally, the flagellum ends in a hard, hollow spine protected by a sheath which opens into the vestibule. The hollow spine is narrow, straight, elongated. Its base is conical. Its diameter is ca. 50 μm except at the base where it is larger (ca. 100 μm). The diameter of the opening at the tip measures ca. 30 μm. Its length ranges from 0.7 mm [1037] (BDMNH) to 1 mm [2693] (MTQ). There is no disc separating the spine of the penial gland and the vestibule.

###### Remarks.

A new species name is needed because no existing name applies to the species described here, based on the examination of all the type specimens available in the Onchidiidae, a careful study of all the original descriptions, and our ongoing taxonomic revision of every genus of the family (Dayrat et al. 2016, 2017, 2018, 2019; Dayrat and Goulding 2017; Goulding et al. 2018a, b, c). Several problematic species names, already discussed in detail in our revision of *Paromoionchis* (Dayrat et al. 2019: 68–72), are regarded as nomina dubia for a variety of reasons (the type locality is too vague, the original description is not informative enough, the type material is destroyed or lost). One of those nomina dubia, *Onchidium palaense* Semper, 1880 (type locality in Aibukit, Palau Islands) could belong to *Paromoionchis* or *Laspionchis* but its generic placement cannot be determined. *Onchidium palaense* does not belong to *Onchidium* because several traits mentioned by Semper, such as the absence of a rectal gland and of an accessory penial gland, are incompatible with *Onchidium* (Dayrat et al. 2016). *Onchidium palaense* simply is a nomen dubium which was arbitrarily placed in the genus *Onchidium* and cannot reliably be placed in any of the onchidiid genera.

##### Laspionchis bourkei

Taxon classificationAnimaliaSystellommatophoraOnchidiidae

Dayrat & Goulding sp. nov.

1CE7ECD7-99A3-5665-BEF7-5BDA51224F74

http://zoobank.org/25128A1E-3115-4EF4-9287-537E18D2F955

Figs 17 , 18 , 19 , 20 , 21 , 22 , 23 , 24 , 25 , 26 , 27 , 28 , 29

###### Holotype.

AUSTRALIA • holotype, designated here, 23/18 mm [1657 H]; Northern Territory, Darwin; 12°33.228'S, 130°52.580'E; 14 Aug. 2012; B Dayrat and party leg.; st 61, on the right side of the road just before bridge to Channel Island, *Avicennia* mangrove with sandy mud; NTM P.57615.

###### Additional material examined.

See below for each subspecies.

###### Distribution

(Fig. 7). Australia (Northern Territory) for *L. bourkei bourkei*. Indonesia (Ambon) for *L. bourkei lateriensis*. Indonesia (Sulawesi, Sumatra), Malaysia (Peninsular Malaysia), Singapore, Philippines (Bohol), and Vietnam for *L. bourkei matangensis*.

###### Habitat

**Figure 17.**
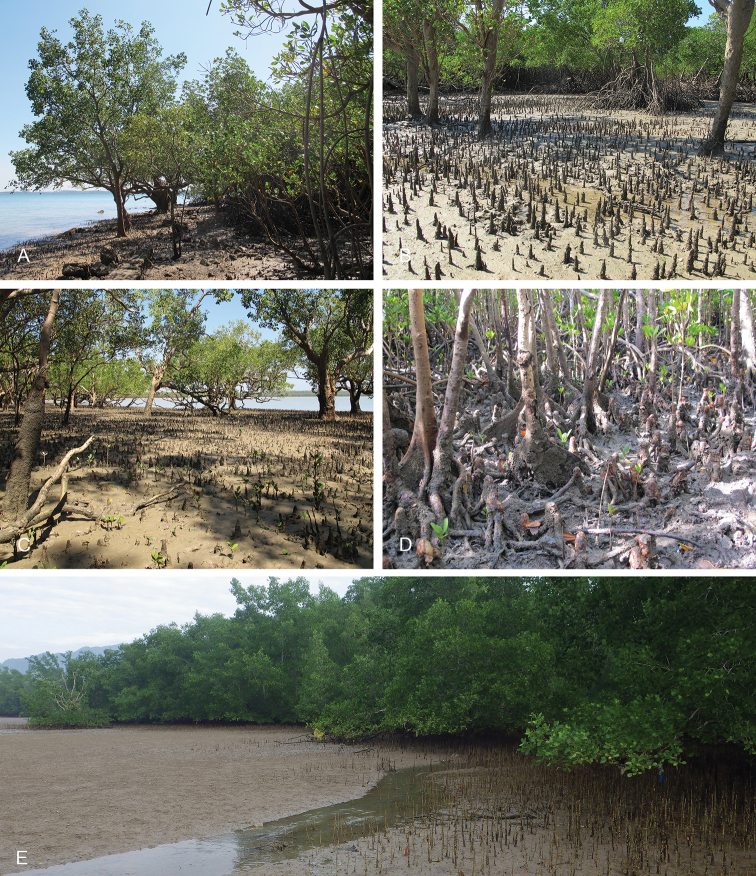
Habitats, *Laspionchis bourkei***A–D***L. bourkei bourkei* Australia, Northern Territory **E***L. bourkei lateriensis*, Indonesia, Ambon. **A***Avicennia* on sandy mud (st 61, type locality) **B** same as **A C** large *Sonneratia alba*, open forest, soft mud by shore (st 62) **D***Sonneratia*, *Rhizophora*, and *Ceriops* mangrove (st 66) **E** mudflat beside a mangrove and a creek (st 128, type locality).

(Figs 17, 26). *Laspionchis bourkei* is found on mud, hard or soft, in open or dense mangrove forests. It can be locally common across its entire distribution.

###### Etymology.

*Laspionchis bourkei* is dedicated to Adam Bourke, from Darwin, Northern Territory, Australia, a very knowledgeable mangrove expert and great naturalist, who generously accompanied us in the field around Darwin and showed us good collecting sites.

###### Diagnosis

(Table 3). Externally, *Laspionchis bourkei* cannot be distinguished from *L. boucheti*. Internally, however, the long retractor muscle of the penis inserts at the posterior end of the visceral cavity in *L. boucheti* while the retractor muscle is short (and inserting in the first third of the visceral cavity) in *L. bourkei bourkei* and vestigial or absent in *L. bourkei lateriensis* and *L. bourkei matangensis*. Also, additional, distal, retractor muscle fibers are present in *L. boucheti* but absent in *L. bourkei*.

###### Color and morphology of live animals

**Figure 18.**
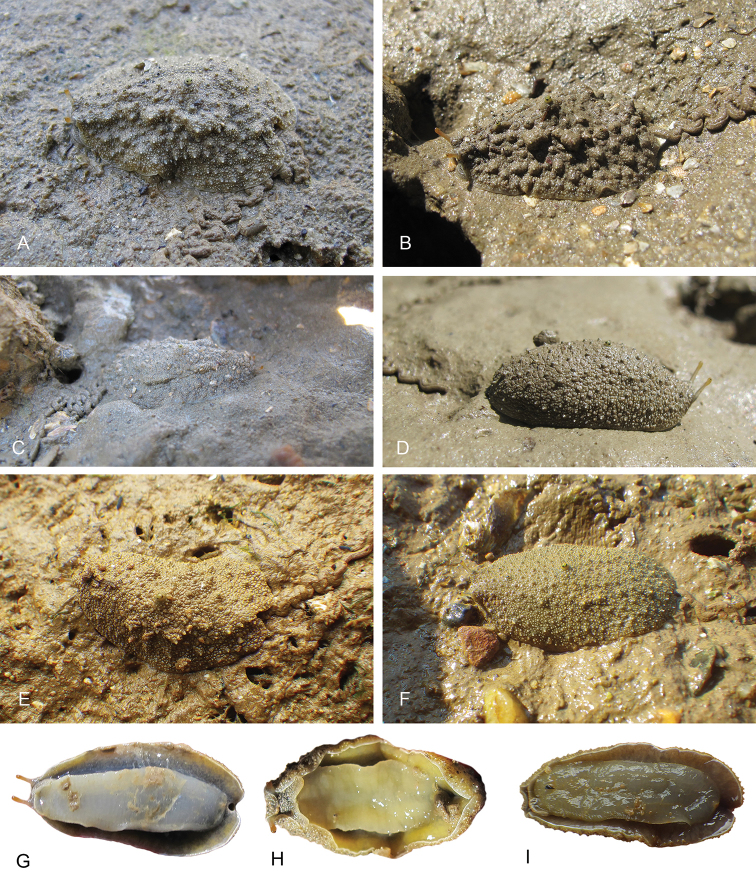
Live animals, *Laspionchis bourkei***A–D, G, H***L. bourkei bourkei* Australia, Northern Territory **E, F, I***L. bourkei lateriensis*, Indonesia, Ambon. **A** Dorsal view, 20 mm long [1618] (NTM P.57617) **B** dorsal view, 21 mm long [1656] (NTM P.57617) **C** holotype, dorsal view, 23 mm long [1657 H] (NTM P.57615) **D** dorsal view, 19 mm long [1673] (NTM P.57618) **E** dorsal view, 12 mm long [2743] (UMIZ 00116) **F** dorsal view, 17 mm long [2753] (UMIZ 00116) **G** ventral view, 19 mm long [1659] (NTM P.57618) **H** ventral view, 23 mm long [1617] (NTM P.57617) **I** ventral view, same as **E**.

(Figs 18, 27). Live slugs are covered with mud and their dorsal color can hardly be seen. The background of the dorsal notum is brown, light to dark, homogenous or mottled with darker or lighter areas. The color of the foot is a mix of gray (light or dark) and yellow, as is the color of the hyponotum. The color of the ventral surface (foot and hyponotum) can change rapidly, especially when slugs are disturbed. The ocular tentacles are brown (variable from light to dark) and short (a few millimeters). The number of papillae with dorsal eyes is variable (between five and ten, on average) and they mostly are on the central part of the notum.

###### Digestive system

**Figure 19.**
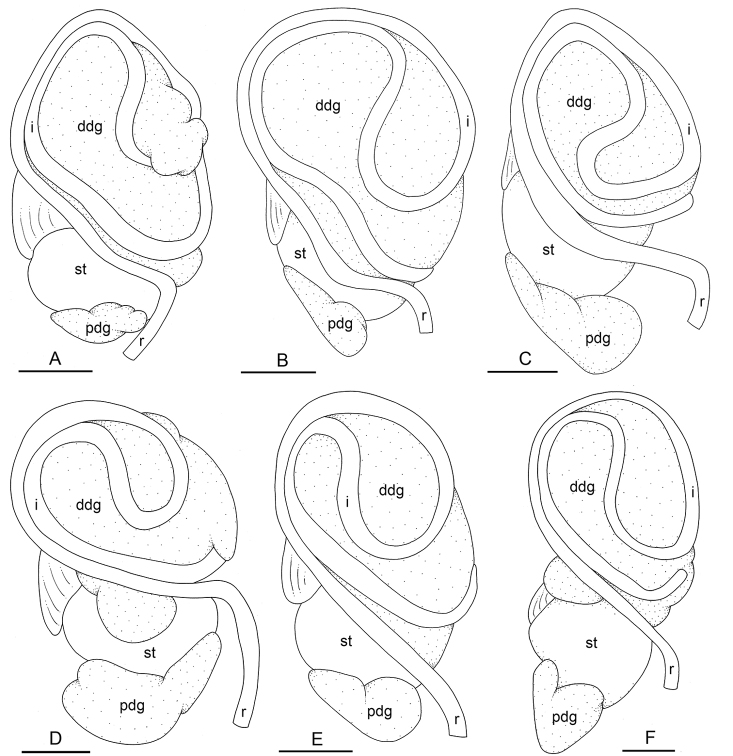
Digestive system, dorsal view, *Laspionchis bourkei***A–C***L. bourkei bourkei*, Australia, Northern Territory **D** Holotype, *L. bourkei lateriensis*, Indonesia, Ambon **E, F***L. bourkei matangensis*, Peninsular Malaysia. **A** [1673] (NTM P.57618) **B** [1666] (NTM P.57618) **C** [1693] (NTM P.57619) **D** [6064 H] (UMIZ 00115) **E** [5959] (USMMC 00056) **F** holotype, [5958 H] (USMMC 00055). Scale bars: 2 mm (**A, C–E**), 2.5 mm (**B**), 1 mm (**F)**. Abbreviations: ddg dorsal digestive gland i intestine pdg posterior digestive gland r rectum st stomach.

**Figure 20.**
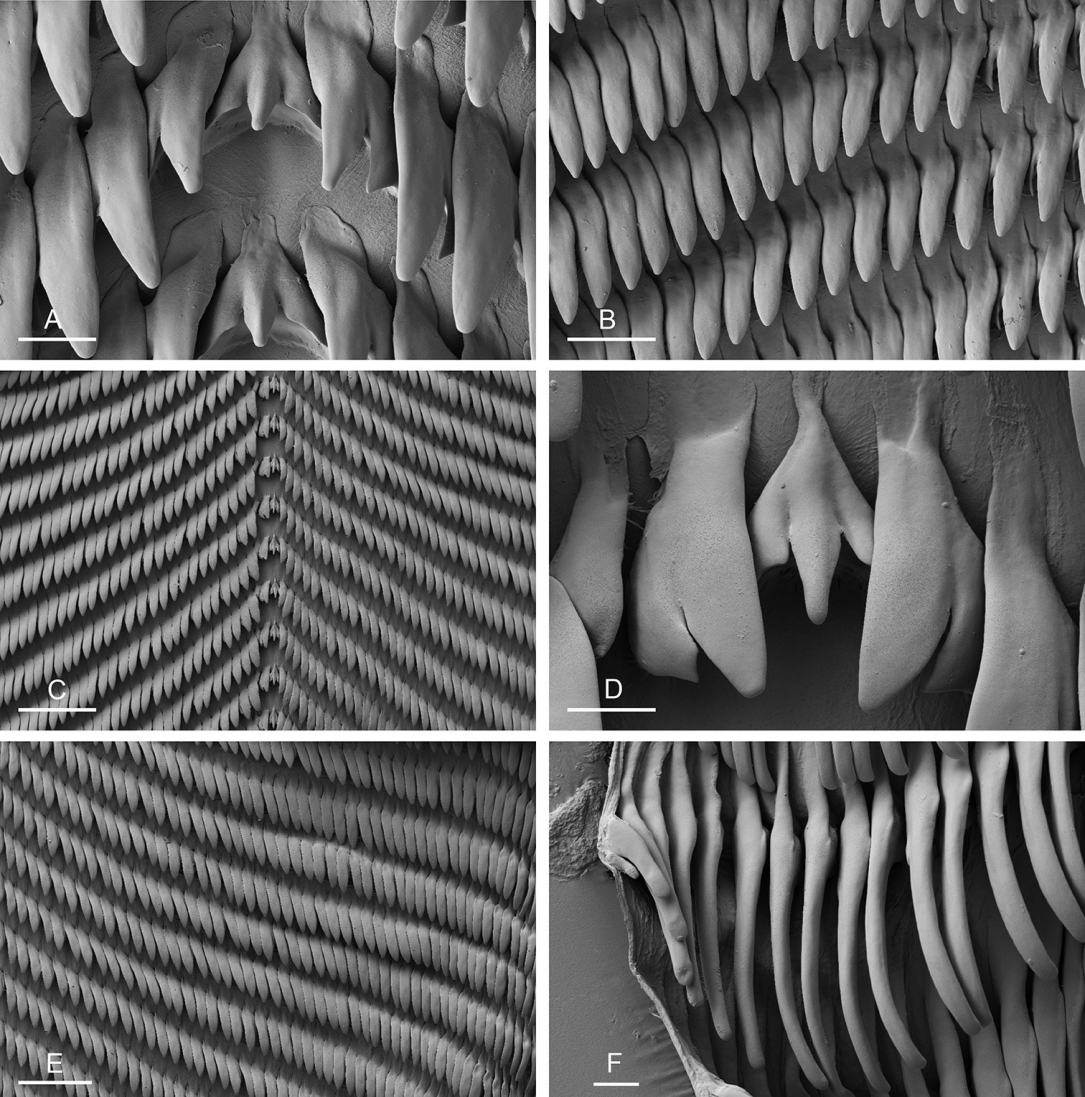
Radula, *Laspionchis bourkei***A, B***L. bourkei matangensis*, Peninsular Malaysia [5965] (USMMC 00056) **C–F** Holotype, *L. bourkei bourkei*, Australia, Northern Territory [1657 H] (NTM P.57615). **A** Rachidian and innermost lateral teeth **B** lateral teeth **C** rachidian and lateral teeth **D** rachidian and innermost lateral teeth **E** lateral teeth **F** outermost lateral teeth. Scale bars: 10 μm (**A, D, F**), 30 μm (**B**), 100 μm (**C**), 80 μm (**E)**.

(Figs 19, 20, 25). Examples of radular formulae are in Table 4. Radulae measure up to 2.9 mm in length (see below for each subspecies). The intestine is long, narrow, and the intestinal loops are exactly between types I and II, i.e., with a transitional loop on average oriented at 6 o’clock, acknowledging minor individual variation (Figs 1, 19).

###### Reproductive system

**Figure 21.**
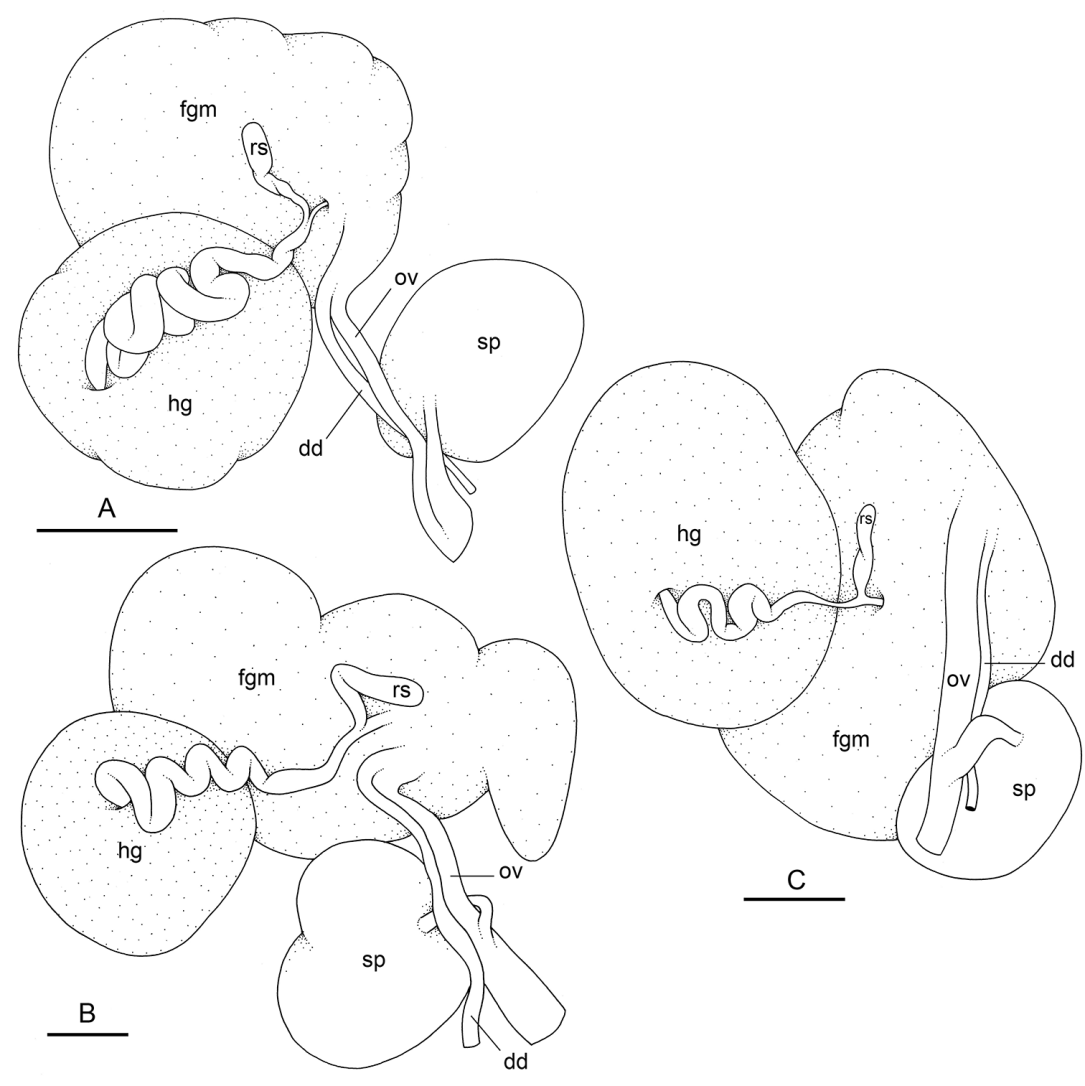
Posterior, hermaphroditic (female) reproductive system, *Laspionchis bourkei*. **A** Holotype, *L. bourkei bourkei*, Australia, Northern Territory, [1657 H] (NTM P.57615) **B** holotype, *L. bourkei lateriensis*, Indonesia, Ambon, [6064 H] (UMIZ 00115) **C** holotype, *L. bourkei matangensis*, Peninsular Malaysia, [5958 H] (USMMC 00055). Scale bars: 2 mm (**A**), 1 mm (**B, C)**. Abbreviations: dd deferent duct fgm female gland mass hg hermaphroditic gland ov oviduct rs receptaculum seminis sp spermatheca.

(Fig. 21). There is a narrow receptaculum seminis (caecum) along the hermaphroditic duct. The large, ovate-spherical spermatheca connects to the oviduct through a narrow and short duct. The oviduct is straight, slightly larger than the deferent duct or of a similar diameter.

###### Copulatory apparatus

**Figure 22.**
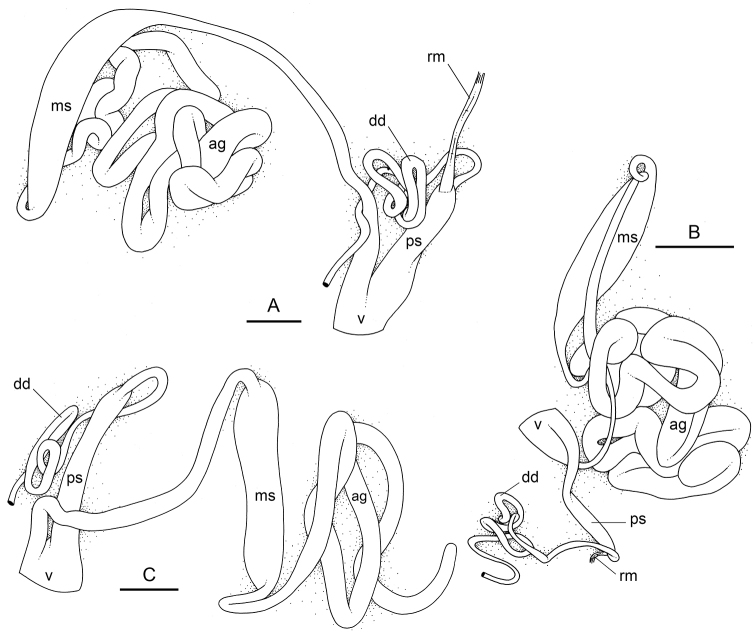
Anterior, male, copulatory apparatus, *Laspionchis bourkei*. **A** Holotype, *L. bourkei bourkei*, Australia, Northern Territory, [1657 H] (NTM P.57615) **B** holotype, *L. bourkei lateriensis*, Indonesia, Ambon, [6064 H] (UMIZ 00115) **C** holotype, *L. bourkei matangensis*, Peninsular Malaysia, [5958 H] (USMMC 00055). Scale bars: 1 mm (**A)**, 2 mm (**B)**, 0.5 mm (**C)**. Abbreviations: ag accessory penial gland dd deferent duct ms muscular sac ps penial sheath rm retractor muscle v vestibule.

**Figure 23.**
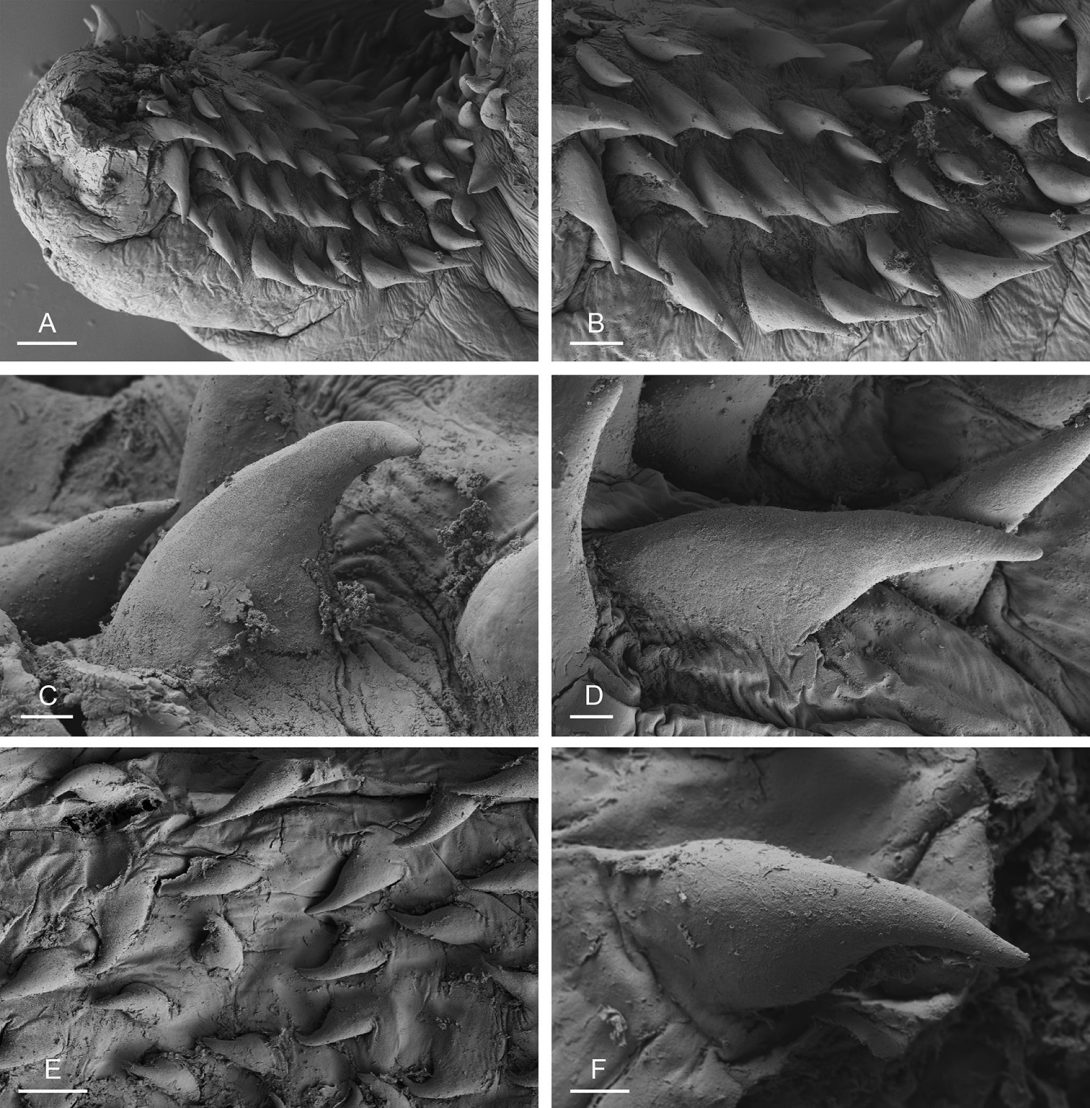
Penial hooks, *Laspionchis bourkei***A–D** Holotype, *L. bourkei lateriensis*, Indonesia, Ambon [6064 H] (UMIZ 00115) **E, F***L. bourkei bourkei*, Australia, Northern Territory [1692] (NTM P.57619). Scale bars: 40 μm (**A, E)**, 20 μm (**B)**, 5 μm (**C, D)**, 4 μm **(F)**.

**Figure 24.**
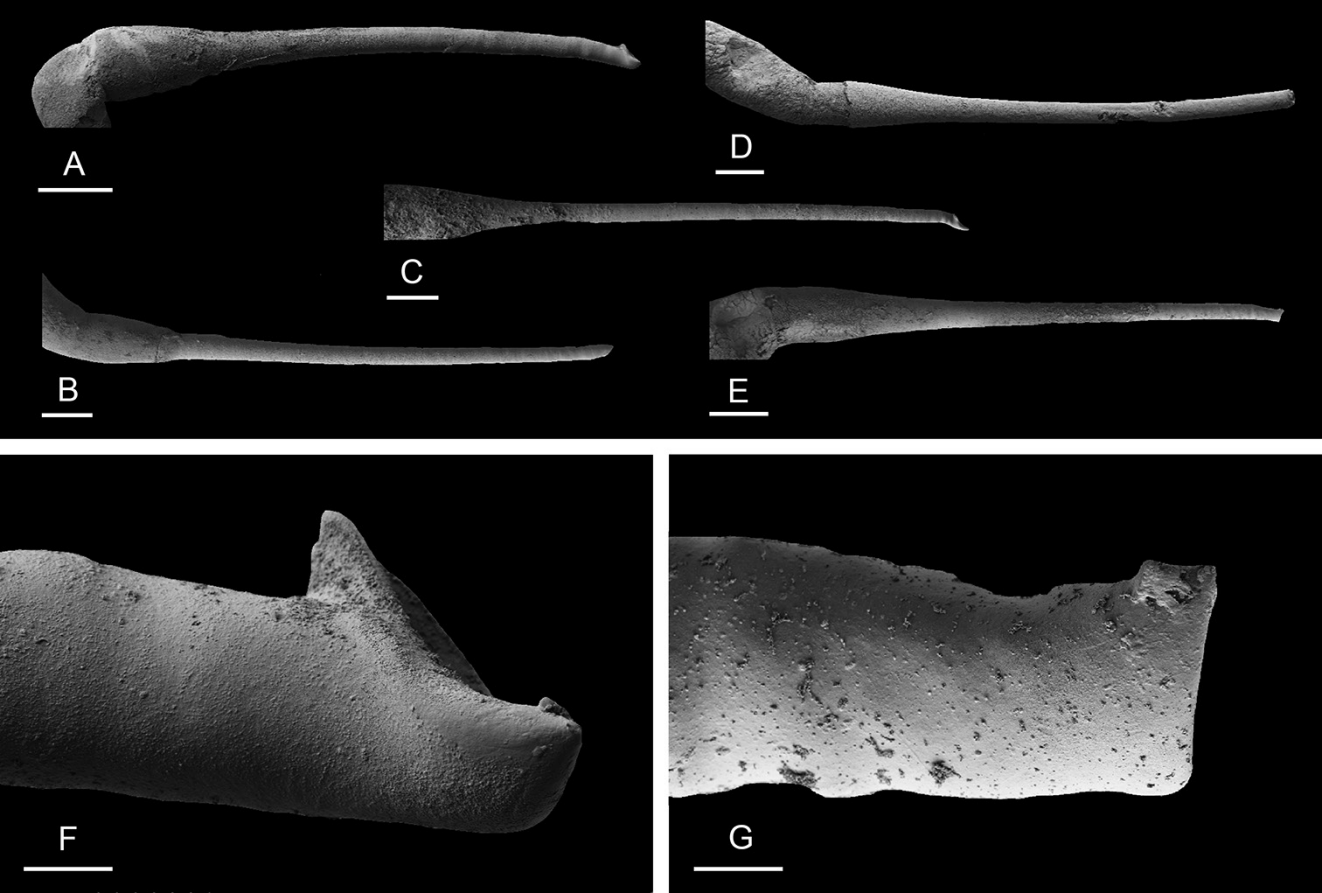
Spine of the accessory penial gland, *Laspionchis bourkei***A–D, F***L. bourkei bourkei*, Australia, Northern Territory **E, G***L. bourkei lateriensis*, Indonesia, Ambon. **A** Holotype, [1657 H] (NTM P.57615) **B** [1666] (NTM P.57618) **C** [1692] (NTM P.57619) **D** [1673] (NTM P.57618) **E** holotype, [6064 H] (UMIZ 00115) **F** same as **A G** same as **E**. Scale bars: 100 μm (**A–E)**, 10 μm (**F, G)**.

(Figs 22–24, 28, 29). The length of the flagellum of the accessory penial gland varies among individuals but it is always heavily coiled. The hollow spine of the penial gland is narrow, straight, elongated. Its base is conical. Its length varies from 0.35 mm to 1 mm (see below for each subspecies). The penial sheath is narrow and short. The penial sheath protects the penis for its entire length. The beginning of the retractor muscle marks the separation between the penial sheath (and the penis inside) and the deferent duct. The retractor muscle is short (as long as the penial sheath) and inserting in the first third of the visceral cavity, vestigial (and free with no attachment), or absent (see below for each subspecies). There is no additional, distal, retractor muscle fibers. The deferent duct is highly convoluted. Inside the penial sheath, the penis is a narrow, thin, elongated, hollow tube, with numerous and densely-arranged (next to each other) hooks in its distal part. Penial hooks are pointed and measure from 15 to 45 μm (see below for each subspecies). When the penis is retracted inside the penial sheath, the hooks are inside the tube-like penis; during copulation, the penis is everted like a glove and the hooks are then on the outside.

###### Remarks.

A new species name is needed because no existing name applies to the species described here, based on the examination of all the type specimens available in the Onchidiidae, a careful study of all the original descriptions, and our ongoing taxonomic revision of each genus of the family (Dayrat et al. 2016, 2017, 2018, 2019; Dayrat and Goulding 2017; Goulding et al. 2018a, b, c).

*Laspionchis bourkei* is divided in three distinct units of which the reciprocal monophyly is highly-supported in both mitochondrial and nuclear analyses (except for *L. bourkei matangensis*, unresolved using nuclear data). The fact that the three units within *L. bourkei* are distinct taxa means that they should be recognized and named. Even though we could have ranked them as species, we decided to rank them as sub-species for three main reasons.

(1) The three units within *L. bourkei* are cryptic externally and internally. Some minor anatomical differences seem to exist but which can hardly be used for identification (Table 3).

(2) Ranking the three units within *L. bourkei* as subspecies rather than species is more in agreement with the genetic distances observed between *L. boucheti* and *L. bourkei*. Indeed, the distance gap between *L. boucheti* and *L. bourkei* is between 2.5% and 7.5%, while the distance gap between the three *L. bourkei* units is between 2.5% and 3.9%, clearly suggesting that the three *L. bourkei* units are much less divergent (their COI sequences) than *L. boucheti* and *L. bourkei*, supporting their ranking as subspecies. Distance values should not necessarily be compared from one genus to another, but they can be compared between very closely related species.

(3) As of today, the three units within *L. bourkei* are allopatric which means that a doubt remains as to whether the three units are reproductively isolated or not. Overall, the three units within *L. bourkei* probably are relatively young taxa which diverged recently, explaining that they are cryptic internally and that their COI sequences are less divergent than the COI sequences between *L. boucheti* and *L. bourkei*.

##### Laspionchis bourkei bourkei

Taxon classificationAnimaliaSystellommatophoraOnchidiidae

Dayrat & Goulding ssp. nov.

FB336EB4-C57A-55F8-AA12-FDB6ADE6AAC1

http://zoobank.org/AEBEFFC9-8AC4-48E1-A37B-5F1E99EFE75D

Figs 17A–D , 18A–D, G, H , 19A–C , 20C–F , 21A , 22A , 23E, F , 24A–D, F

###### Holotype.

The type locality and the holotype of the nominotypical subspecies *L. bourkei bourkei* are the same as those of the nominal species *L. bourkei* (ICZN Arts. 47.1, 61.2, and 72.8).

###### Additional material examined.

AUSTRALIA – **Northern Territory** • 1 specimen 8/4 mm [1616]; Darwin, Lee Point Road, Buffalo Creek; 12°20.460'S, 130°54.600'E; 13 Aug. 2012; B Dayrat and party leg.; st 60, narrow *Rhizophora* mangrove by a river with very dry and hard mud NTM P.57616. • 4 specimens 20/12 mm [1652], 21/13 mm [1656], 23/15 mm [1617], 20/15 mm [1618]; same collection data as for the holotype; NTM P.57617. • 4 specimens 18/10 mm [1621], 19/12 mm [1659], 32/20 mm [1666], 19/12 mm [1673]; Darwin, Talc Head; 12°28.765'S, 130°46.297'E; 15 Aug. 2012; B Dayrat and party leg.; st 62, large and open forest of *Sonneratia alba* with soft mud; NTM P.57618. • 3 specimens 25/18 mm [1692], 22/15 mm [1693], 22/15 mm [1694]; Darwin, end of the Channel Island Road; 12°33.557'S, 130°52.894'E; 17 Aug. 2012; B Dayrat and party leg.; st 66, sequence of *Sonneratia*, *Rhizophora*, and *Ceriops*; NTM P.57619.

###### Distribution

(Fig. 7). Australia (Northern Territory).

###### Habitat

(Fig. 17A–D). Same as the entire species *Laspionchis bourkei* (see above).

###### Etymology.

See above, the species *L. bourkei*.

###### Diagnosis

(Table 3). Externally, the three subspecies of *L. bourkei* cannot be distinguished. Internally, *L. bourkei bourkei* differs from both *L. bourkei lateriensis* and *L. bourkei matangensis*. Indeed, *L. bourkei bourkei* is characterized by a short retractor muscle of the penis which inserts in the anterior third of the visceral cavity while the retractor muscle is vestigial or absent in *L. bourkei lateriensis* and *L. bourkei matangensis*. Also, the spine of the accessory penial gland is on average slightly longer in *L. bourkei bourkei* than in *L. bourkei lateriensis* and *L. bourkei matangensis*.

###### Color and morphology of live animals

(Fig. 18A–D, G, H). Identical to the species *L. bourkei* (see above).

###### Digestive system

(Figs 19A–C, 20C–F). Identical to the species *L. bourkei* (see above). Examples of radular formulae are in Table 4. Radulae measure up to 2.9 mm in length.

###### Reproductive system

(Fig. 21A). Identical to the species *L. bourkei* (see above).

###### Copulatory apparatus

(Figs 22A, 23E, F, 24A–D, F). Similar to the species *L. bourkei* (see above) acknowledging some minor variations: the length of the spine of the accessory penial gland ranges from 0.75 mm [1657 H] (NTM P.57615) to 1 mm [1666] (NTM P.57618), the retractor muscle is short (as long as the penial sheath) and inserts in the first third of the visceral cavity, and penial hooks measure from 20 to 35 μm.

###### Remarks.

See above, the remarks on the species *Laspionchis bourkei*.

##### Laspionchis bourkei lateriensis

Taxon classificationAnimaliaSystellommatophoraOnchidiidae

Dayrat & Goulding ssp. nov.

871AA8E8-70C1-5456-A1DD-235273B99F88

http://zoobank.org/05FE1E0F-0679-4AA0-A40B-21FBE2D1199B

Figs 17E , 18E, F, I , 19D , 21B , 22B , 23A–D , 24E, G , 25

###### Holotype.

INDONESIA • holotype, designated here, 17/16 mm [6064 H]; Ambon, Lateri; 03°38.261'S, 128°14.716'E; 12 Feb. 2014; M Khalil and party leg.; st 128, mudflat next to small creek in the low intertidal of mangrove preserve; UMIZ 00115.

###### Additional material examined.

INDONESIA – **Ambon** • 5 specimens 12/7 mm [2743], 17/10 mm [2753], 15/12 mm [6061], 18/13 mm [6063], 15/12 mm [6065]; same collection data as for the holotype; UMIZ 00116.

###### Distribution

(Fig. 7). Indonesia (Ambon).

###### Habitat

(Fig. 17E). Same as the entire species *Laspionchis bourkei* (see above).

###### Etymology.

The subspecies *Laspionchis bourkei lateriensis* is named after Lateri, in Ambon because the type locality is part of the preserved mangrove of Lateri. The name *lateriensis* is an adjective derived from Lateri and the suffix -*ensis*.

###### Diagnosis

(Table 3). Externally, the three subspecies of *L. bourkei* cannot be distinguished. Internally, *L. bourkei lateriensis* differs from *L. bourkei bourkei* but cannot be distinguished from *L. bourkei matangensis*. All three subspecies, however, are clearly delineated using molecular DNA sequences.

###### Color and morphology of live animals

(Fig. 18E, F, I). Identical to the species *L. bourkei* (see above).

###### Digestive system

**Figure 25.**
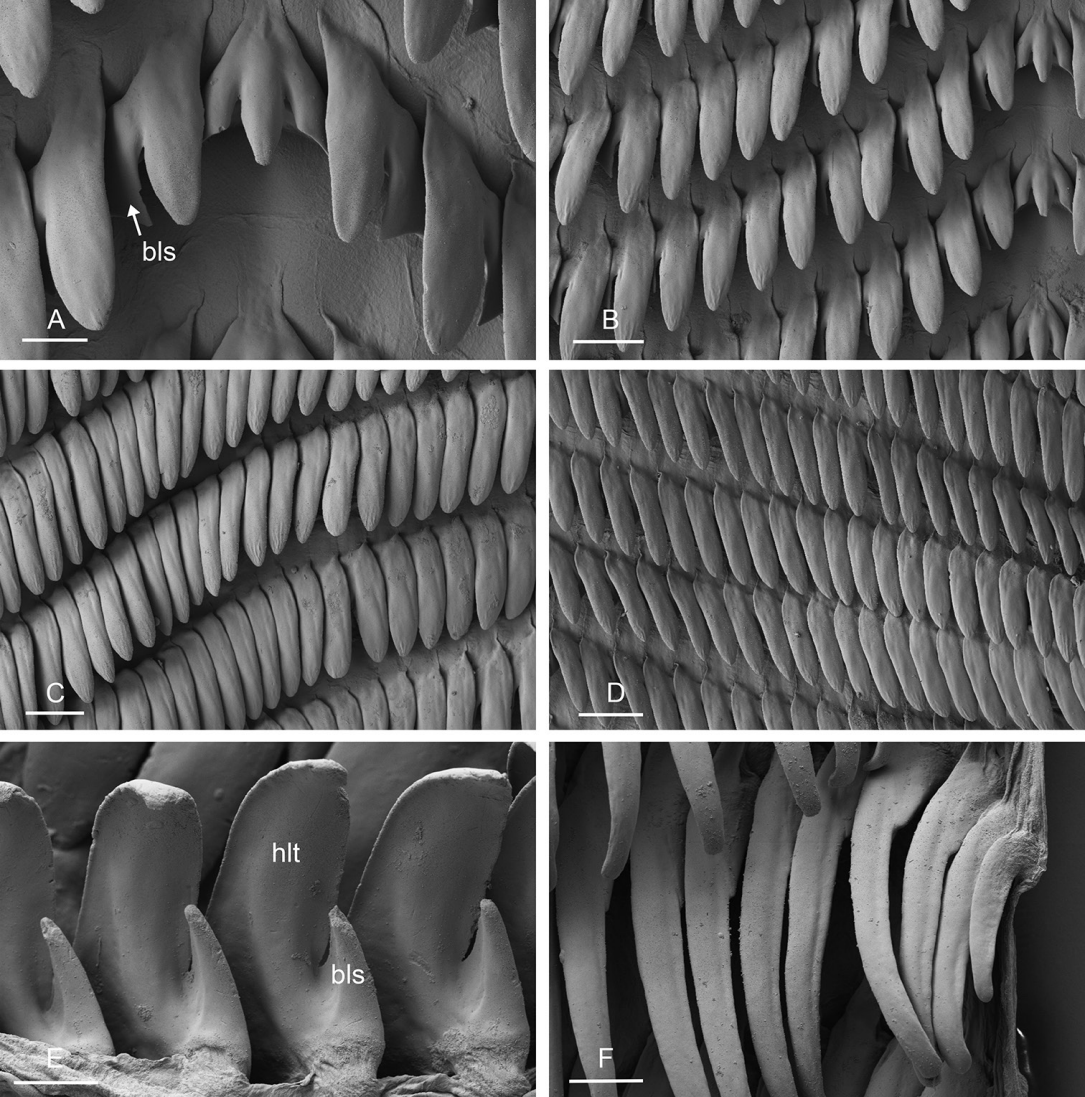
Radula, *Laspionchis bourkei lateriensis*, Indonesia, Ambon **A–D** holotype [6064 H] (UMIZ 00115) **E, F** [6063] (UMIZ 00116). **A** Rachidian and innermost lateral teeth **B** rachidian and innermost lateral teeth **C** lateral teeth **D** lateral teeth **E** lateral teeth (inferior view) **F** outermost lateral teeth. Scale bars: 10 μm (**A, F)**, 20 μm (**B, C)**, 30 μm (**D)**, 8 μm (**E)**. Abbreviations: bls basal lateral spine hlt hook of a lateral tooth.

(Figs 19D, 25). Identical to the species *L. bourkei* (see above). Examples of radular formulae are presented in Table 4. Radulae measure up to 1.7 mm in length.

###### Reproductive system

(Fig. 21B). Identical to the species *L. bourkei* (see above).

###### Copulatory apparatus

(Figs 22B, 23A–D, 24E, G). Similar to the species *L. bourkei* (see above) acknowledging some minor variations: the length of the spine of the accessory penial gland ranges from 0.35 mm [6063] (UMIZ 00116) to 0.75 mm [6064 H] (UMIZ 00115), the retractor muscle is vestigial or absent, and penial hooks measure from 20 to 45 μm.

###### Remarks.

See above, the remarks on the species *Laspionchis bourkei*.

##### Laspionchis bourkei matangensis

Taxon classificationAnimaliaSystellommatophoraOnchidiidae

Dayrat & Goulding ssp. nov.

859D67DB-3B7E-54FF-AC38-E497BE07BB94

http://zoobank.org/05BA0236-4FFB-4F2E-BDB2-58BFECE43FFC

Figs 19E, F , 20A, B , 21C , 22C , 26 , 27 , 28 , 29

###### Holotype.

MALAYSIA • holotype, designated here, 15/9 mm [5958 H]; Peninsular Malaysia, Matang, facing fishermen’s village on the other side of river; 04°50.217'N, 100°36.826'E; 26 Jul. 2016; B Dayrat and party leg.; st 256, oldest and open *Rhizophora* forest of tall and beautiful trees, with hard mud, many creeks, and many old logs; USMMC 00055.

###### Additional material examined.

INDONESIA – **Sumatra** • 3 specimens 11/7 mm [1783], 10/7 mm [1784], 13/9 [1785]; Tembilahan; 00°10.243'S, 103°27.982'E; 13 Oct. 2012; M Khalil and party leg.; st 76, mangrove of large *Avicennia* trees, with old logs, soft but solid mud, and *Nypa* on the margin; UMIZ 00113. – **Sulawesi** • 1 specimen 12/10 mm [2230]; Bahoi; 01°43.355'N, 125°01.232'E; 12 Mar. 2013; M Khalil and party leg.; st 88, sand, small rocks, pieces of wood outside narrow coastal mangrove; UMIZ 00114. MALAYSIA – **Peninsular Malaysia** • 5 specimens 15/8 mm [5959], 12/9 mm [5960], 15/8 mm [5961], 13/8 mm [5963], 8/5 mm [5965]; same collection data as for the holotype; USMMC 00056. PHILIPPINES – **Bohol** • 1 specimen 25/18 mm [3343]; Mabini; 09°51.532'N, 124°31.685'E; 17 Jul. 2014; B Dayrat and party leg.; st 194, narrow mangrove on the edge of fish ponds, tall *Rhizophora* and *Avicennia* trees, many old logs; PNM 041254. • 1 specimen 10/7 mm [3616]; Inabanga; 10°00.389'N, 124°03.522'E; 12 Jul. 2014; B Dayrat and party leg.; st 186, old, rehabilitated fish ponds next to a mangrove with some old *Avicennia* but mostly young *Rhizophora* trees; PNM 041253. SINGAPORE • 3 specimens 9/7 mm [978], 9/8 mm [979], 10/8 mm [980]; Lim Chu Kang; 01°26.785'N, 103°42.531'E; 2 Apr. 2010; B Dayrat and party leg.; st 7, east of the jetty, open mangrove with medium trees, ending on mudflat outside mangrove with soft mud; ZRC.MOL.10485. • 2 specimens 13/8 mm [983], 10/7 mm [985]; Mandai River; 01°26.237'N, 103°45.730'E; 1 Apr. 2010; B Dayrat and party leg.; st 6, open mangrove forest with tall trees and soft mud, ending on mudflat outside the mangrove with very soft mud; ZRC.MOL.10484. VIETNAM • 2 specimens 8/4 mm [5627], 4/3 mm [5646]; Can Gio; 10°24.171'N, 106°53.960'E; 10 Jul. 2015; TC Goulding and party leg.; st 221, open *Avicennia* and *Rhizophora* mangrove with hard mud by a small road and deep mud near water; ITBZC IM 00018.

###### Distribution

(Fig. 7). Indonesia (Sulawesi, Sumatra), Malaysia (Peninsular Malaysia), Singapore, Philippines (Bohol), Vietnam.

###### Habitat

**Figure 26.**
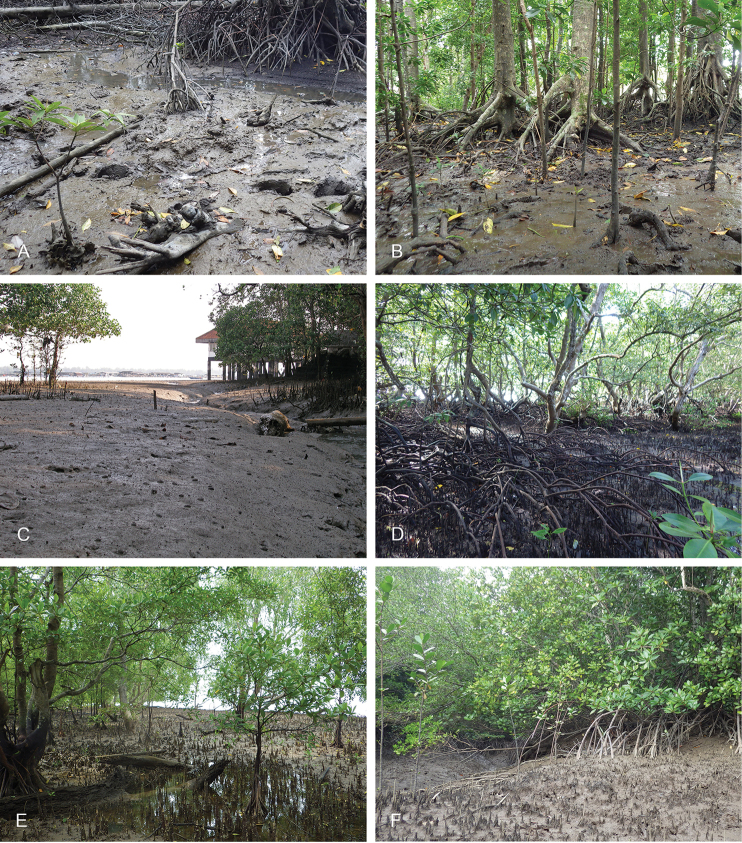
Habitats, *Laspionchis bourkei matangensis*. **A, B** Peninsular Malaysia, Matang, old and open *Rhizophora* forest of with hard and soft mud, many creeks, and many old logs (st 256, type locality) **C** Singapore, mud outside mangrove on sun-exposed mudflat (st 7) **D** Philippines, narrow mangrove on the edge of fish ponds, tall *Rhizophora* and *Avicennia* trees, many old logs (st 194) **E** Indonesia, Sumatra, soft but solid mud, big *Avicennia*, a few logs, some *Nypa* on margin, little open space (st 76) **F** Vietnam, open *Avicennia* and *Rhizophora* mangrove with hard mud by a small road and deep mud near water (st 221).

(Fig. 26). Same as the entire species *Laspionchis bourkei* (see above).

###### Etymology.

The subspecies *L. bourkei matangensis* is named after Matang, in Peninsular Malaysia. The type locality is part of the Matang mangrove forest. The name *matangensis* is an adjective derived from Matang and the suffix -*ensis*.

###### Diagnosis

(Table 3). Externally, the three subspecies of *L. bourkei* cannot be distinguished. Internally, *L. bourkei matangensis* differs from *L. bourkei bourkei* but cannot be distinguished from *L. bourkei lateriensis*. All three subspecies, however, are clearly delineated using molecular DNA sequences.

###### Color and morphology of live animals

**Figure 27.**
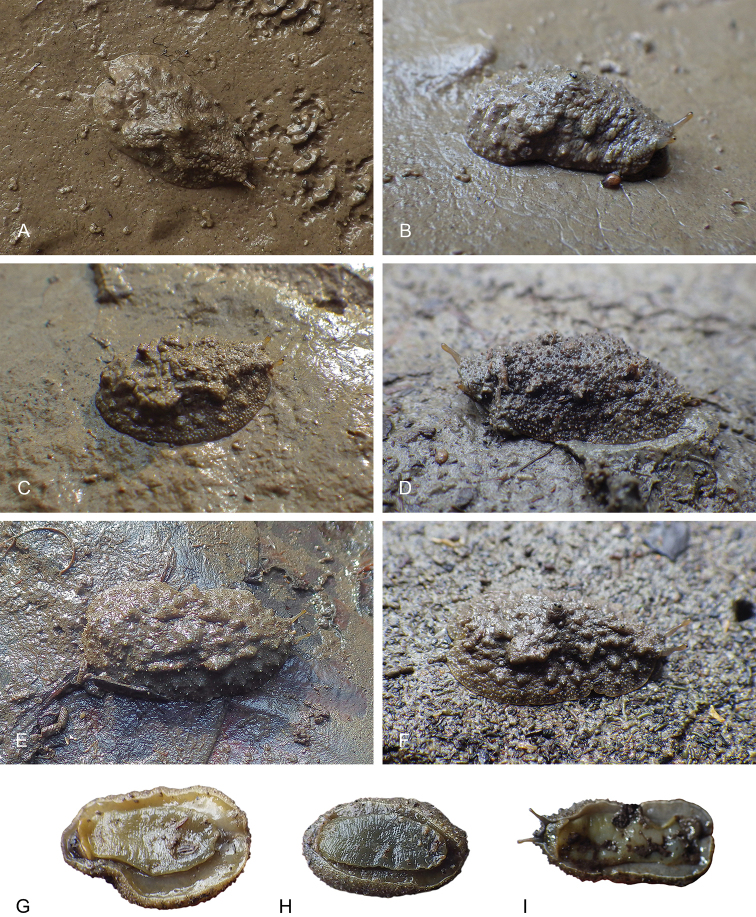
Live specimens, *Laspionchis bourkei matangensis*. **A** Dorsal view, 13 mm long [1785], Indonesia, Sumatra (UMIZ 00113) **B** dorsal view, 10 mm long [1784], Indonesia, Sumatra (UMIZ 00113) **C** dorsal view, 11 mm long [1783], Indonesia, Sumatra (UMIZ 00113) **D** dorsal view, 15 mm long [5961], Peninsular Malaysia, Matang (USMMC 00056) **E** dorsal view, 25 mm long [3343], Philippines, Bohol (PNM 041254) **F** holotype, dorsal view, 15 mm long [5958 H], Peninsular Malaysia, Matang (USMMC 00055) **G** ventral view, 13 mm long [5963], Peninsular Malaysia, Matang (USMMC 00056) **H** ventral view, 8 mm long [5965], Peninsular Malaysia, Matang (USMMC 00056) **I** ventral view, same as **E**.

(Fig. 27). Identical to the species *L. bourkei* (see above).

###### Digestive system

(Figs 19E, F, 20A, B). Identical to the species *L. bourkei* (see above). Examples of radular formulae are in Table 4. Radulae measure up to 2.2 mm in length.

###### Reproductive system

(Fig. 21C). Identical to the species *L. bourkei* (see above).

###### Copulatory apparatus

**Figure 28.**
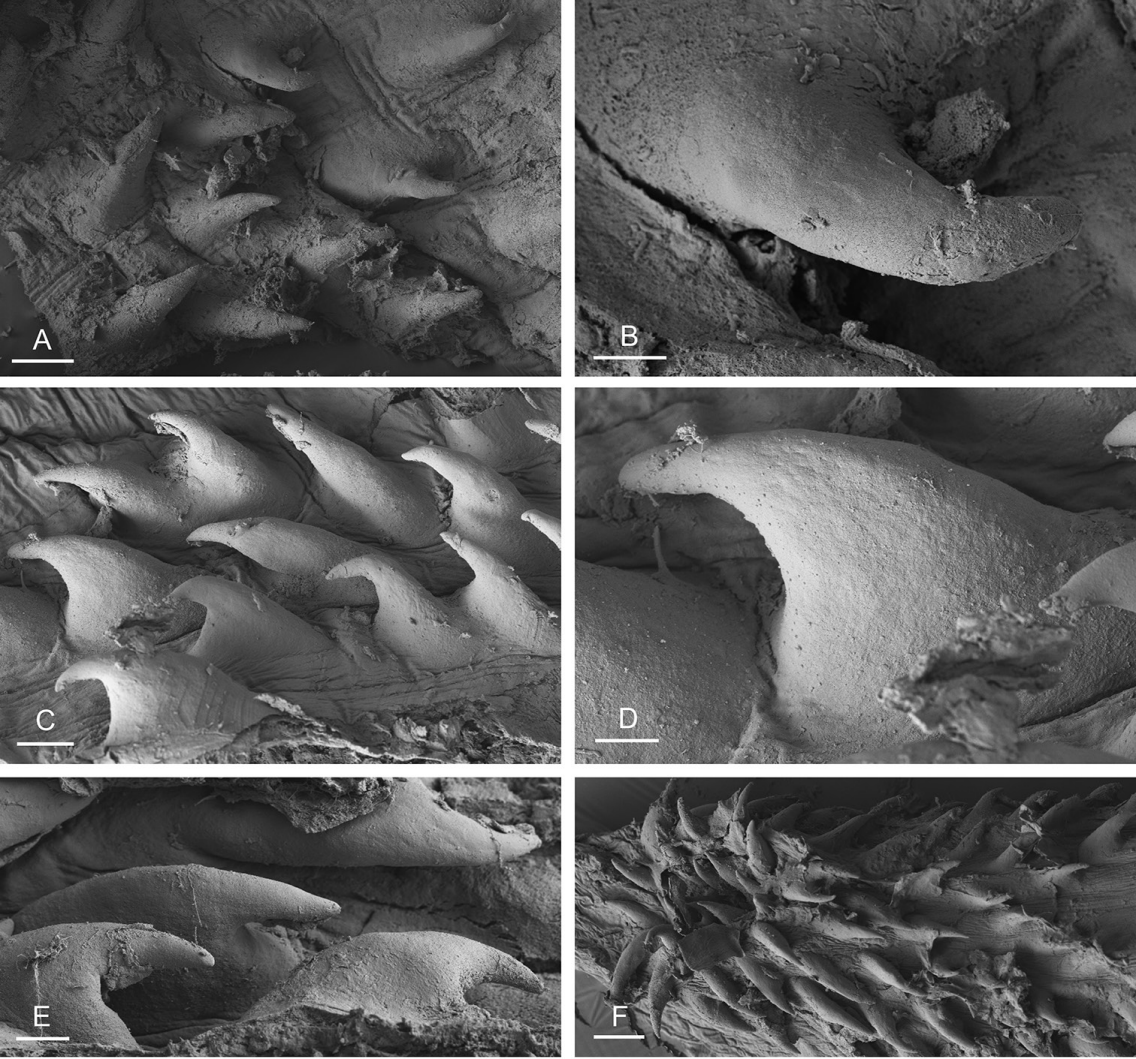
Penial hooks, *Laspionchis bourkei matangensis*, Peninsular Malaysia **A, B** Holotype [5958 H] (USMMC 00055) **C–E** [5959] (USMMC 00056) **F** [5960] (USMMC 00056). Scale bars: 10 μm (**A, C)**, 3 μm (**B)**, 4 μm (**D)**, 5 μm (**E)**, 20 μm (**F)**.

**Figure 29.**
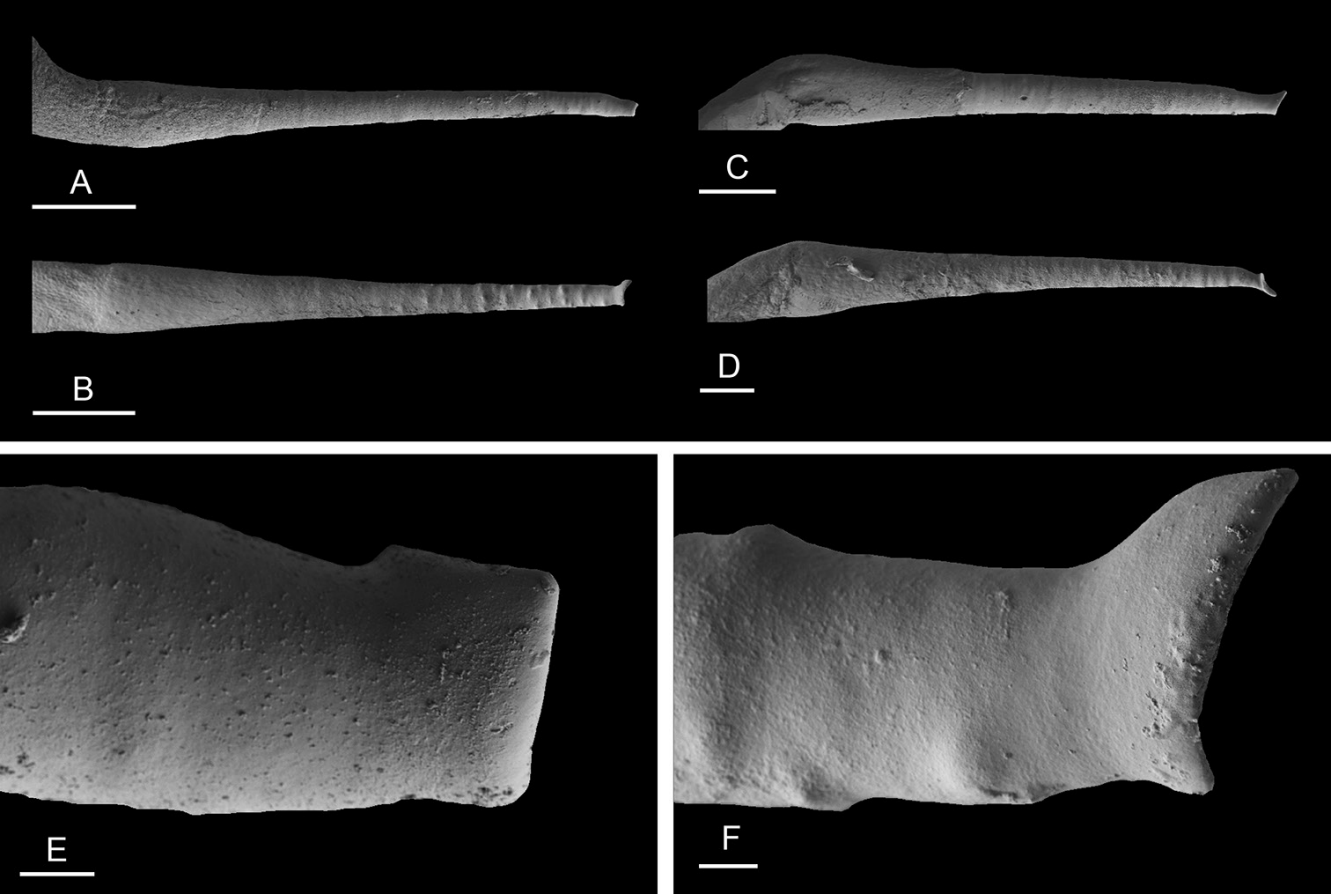
Spine of the accessory penial gland, *Laspionchis bourkei matangensis*. **A** Holotype, Peninsular Malaysia, [5958 H] (USMMC 00055) **B** Peninsular Malaysia, [5959] (USMMC 00056) **C** Philippines, [3343] (PNM 041254) **D** Peninsular Malaysia, [5960] (USMMC 00056) **E** same as **A F** same as **B**. Scale bars: 100 μm (**A–C)**, 50 μm (**D)**, 5 μm (**E, F)**.

(Figs 22C, 28, 29). Similar to the species *L. bourkei* (see above) acknowledging some minor variations: the length of the spine of the accessory penial gland ranges from 0.4 mm [5958 H] (USMMC 00055) to 0.57 mm [3343] (PNM 041254), the retractor muscle is vestigial or absent, and penial hooks measure 15 to 40 μm.

###### Remarks.

See above the remarks on the species *Laspionchis bourkei*.

## Discussion

A few preliminary remarks can be made here regarding the types of intestinal loops, even though a more detailed discussion will be provided after our revisions of *Peronia* and *Platevindex* are published (in preparation).

(1) Nearly all onchidiid species are characterized by only one intestinal type. Some intra-specific variation exists, which can be evaluated based on the orientation of the transitional loop. However, the presence of more than one intestinal type in an onchidiid species remains exceptional, such as in *Alionchis jailoloensis* (see Goulding et al. 2018b).

(2) Nearly all onchidiid genera are characterized by only one or two intestinal types. For instance, *Wallaconchis* and *Marmaronchis* are characterized by intestinal loops of type I; *Onchidina*, *Paromoionchis*, and *Peronina* by type II; *Laspionchis* by loops between types I and II; *Alionchis*, *Melayonchis*, and *Onchidium* by types II and III. *Platevindex* is the only genus characterized by intestinal loops of more than two types (I, II, and III).

(3) Intestinal loops are quite useful to identify genera. For instance, all known species of *Wallaconchis* are characterized by intestinal loops of type I. Therefore, a slug with intestinal loops of type II cannot belong to *Wallaconchis*, unless intestinal loops of type II are found in the future in a new species of *Wallaconchis*. Also, *Laspionchis* slugs are the only ones known so far with an intestinal type between types I and II, with a transitional loop oriented at 6 o’clock (acknowledging individual variation). Therefore, slugs with intestinal loops between types I and II likely belong to *Laspionchis*.

(4) There must be some reasons explaining why intestinal types are not randomly distributed across onchidiid species and genera; however, the exact reasons are still unclear at this stage. Evolutionary history is possibly involved. For instance, the fact that all *Wallaconchis* species are characterized by intestinal loops of type I may be due to the presence of a type I in their common ancestor. Adaptation to different habitats is likely involved as well and will be discussed after our revisions of *Peronia* and *Platevindex* are published (in preparation).

Onchidiids are notoriously difficult to identify, both at the genus and species levels. *Laspionchis* slugs are no exception. They are most readily identified at the genus level using DNA sequences. Externally, they are practically impossible to distinguish from *Paromoionchis* slugs which live in the same habitat (mangrove mud surface) and are often found at exactly the same sites (see Dayrat et al. 2019). The male opening is clearly to the left of the right ocular tentacle in *Paromoionchis*, while it is just below the right ocular tentacle or only slightly to its left in *Laspionchis*, but this character is nearly impossible to check in the field when slugs are alive (because they retract as soon as they are being touched). Animal size can help distinguish *Laspionchis* slugs from *Paromoionchis* slugs in the field. Indeed, the maximum size of *Paromoionchis* slugs – 55 mm in *P. tumidus*, 65 mm in *P. daemelii*, 47 mm in *P. boholensis* Dayrat & Goulding in Dayrat et al. 2019, 48 mm in *P. penangensis* Dayrat & Goulding in Dayrat et al. 2019, and 35 mm in *P. goslineri* Dayrat & Goulding in Dayrat et al. 2019 – is much higher than the maximum size of *Laspionchis* slugs, 31 mm in *L. boucheti* and 32 mm in *L. bourkei*. That being said, animal length needs to be used with caution and it obviously is useless for all individuals less than 30 mm long.

*Laspionchis* is characterized by a unique combination of external and internal traits: no dorsal gills, male opening below the right eye tentacle (or slightly to its left), no rectal gland, intestinal loops between types I and II, accessory penial gland present with a muscular sac, penis with numerous, pointed hooks densely arranged next to each other. This unique combination of characters of *Laspionchis* is close to that of *Paromoionchis* (Dayrat et al. 2019: 19). However, there are three important differences: in *Paromoionchis*, the male opening is clearly to the left of the right ocular tentacle, the intestinal loops are clearly of type II, and penial hooks (which are present in *P. tumidus* but are absent in the four other *Paromoionchis* species) are sparse (i.e., not densely arranged, next to each other) and not pointed (Dayrat et al. 2019: figs 21, 22).

The two known species of *Laspionchis* are cryptic externally but distinct internally. They are found in exactly the same habitats and cannot be distinguished in the field. However, they can be identified successfully with both DNA sequences and internal anatomy. Species externally cryptic but internally distinct have also been observed in *Paromoionchis*, *Peronina*, and *Wallaconchis* (Dayrat et al. 2019; Goulding et al. 2018a, c). In *Marmaronchis*, species are cryptic externally and internally (Dayrat et al. 2018). In *Onchidium* and *Melayonchis*, species are distinct both externally and internally (Dayrat et al. 2016, 2017). Finally, *Alionchis* and *Onchidina* are monotypic, at least as of today (Dayrat and Goulding 2017; Dayrat et al. 2018).

The two new species described here are widespread and can be locally common. That they are discovered only now is not so surprising. *Laspionchis* is restricted to mangroves and mangroves of South-East Asia have been poorly explored and the biodiversity they host remains poorly known. Also, onchidiid taxonomy has been confused for a long time (Dayrat 2009). Now that onchidiid systematics is finally being revised, new taxa are being discovered (e.g., Dayrat et al. 2017; Goulding et al. 2018a, b, c), contributing to a better knowledge of the diversity of mangrove invertebrates in South-East Asia. It is very possible that additional species of *Laspionchis* will be discovered in the future, within or outside its current distribution. However, the study of the biodiversity of *Laspionchis* remains challenging, mostly because *Laspionchis* slugs are very hard to recognize in the field (they are not distinguishable from *Paromoionchis* slugs) and because *Laspionchis* species are externally cryptic (which means that many individuals looking similar need to be collected and individually numbered).

## Supplementary Material

XML Treatment for Laspionchis

XML Treatment for Laspionchis boucheti

XML Treatment for Laspionchis bourkei

XML Treatment for Laspionchis bourkei bourkei

XML Treatment for Laspionchis bourkei lateriensis

XML Treatment for Laspionchis bourkei matangensis
